# Comparative evaluation of two high-throughput qPCR systems for environmental and clinical microbiology applications

**DOI:** 10.1128/aem.01222-26

**Published:** 2026-09-25

**Authors:** Hao Wang, Satoshi Ishii

**Affiliations:** 1 BioTechnology Institute, University of Minnesota 25793, St. Paul, Minnesota, USA; 2 Department of Soil, Water, and Climate, University of Minnesota 311834, St. Paul, Minnesota, USA; Michigan State University, East Lansing, Michigan, USA

**Keywords:** high-throughput quantitative PCR, Biomark, SmartChip, microbial source tracking, antibiotic resistance genes, pre-amplification

## Abstract

High-throughput quantitative PCR (HT-qPCR) is useful for the simultaneous quantification of various genes, such as microbial source tracking (MST) markers, fecal indicator bacteria, pathogens, and antibiotic resistance genes (ARGs) for environmental and clinical studies. While multiple HT-qPCR platforms are commercially available, their performance has rarely been directly compared. In this study, we evaluated and compared two commonly used HT-qPCR platforms, the Takara Bio SmartChip and the Standard BioTools Biomark HD system, using TaqMan probe-based MST assays and intercalating dye-based ARG assays. Despite differences in sample preparation workflows and reaction volumes, the two platforms produced the same detection results for 94.3% of MST sample × assay combinations, and 87.4% of ARG sample × assay combinations. Because HT-qPCR reactions are performed at nanoliter volumes (6.7–100 nL), a specific target amplification (STA) step is often used to improve the detection of low-abundance targets. Comparison of samples before and after STA showed that STA substantially increased detection frequencies on the Biomark HD platform, but had little effect on the SmartChip platform. Although most assays showed strong agreement between pre- and post-STA measurements and between platforms, discrepancies were observed for a subset of assays. Conventional qPCR and digital PCR results indicated that assay-specific amplification efficiency and sample quality contributed to the observed differences. Overall, these results demonstrate that both HT-qPCR platforms can provide reliable quantitative measurements when properly validated. However, consumable costs, workflow complexity, and maintenance requirements differed between platforms. Therefore, platform selection should consider sample throughput, assay requirements, available resources, and study objectives.

**IMPORTANCE:**

High-throughput qPCR is a powerful tool to detect and quantify various genes simultaneously. Numerous studies have used HT-qPCR for environmental and clinical microbiology applications; however, it is unclear whether different HT-qPCR platforms (e.g., Biomark and SmartChip) produce comparable results or not, as the HT-qPCR protocols are platform-specific. In particular, the use of pre-amplification reaction, also known as specific target amplification (STA), varies across studies. This study shows that both Biomark and SmartChip platforms provide similar quantitative data for most assays and that the STA reaction is useful to improve the detection of low-abundance targets without major biases. To accurately quantify on HT-qPCR platforms, assays should be carefully selected and validated. To guide current and future users, this study provides a general framework for assay selection in HT-qPCR.

## INTRODUCTION

Quantitative PCR (qPCR) is commonly used for the detection and quantification of pathogens, antibiotic resistance genes (ARGs), and other genes of interest in various samples, such as water, food, stool, and other clinical and environmental samples (1–3). qPCR can provide sensitive and specific quantitative information that is useful for various applications (4). Based on the chemistry to produce fluorescence signals, qPCR assays are divided into two groups: probe-based (e.g., TaqMan probe) and intercalating dye-based assays (e.g., SYBR Green, EvaGreen) (5). Probe-based assays are generally more sensitive and specific than intercalating dye-based assays, although they require additional costs for fluorophore-labeled probes (6). By using TaqMan probes labeled with different fluorophores, it is also possible to detect multiple targets (i.e., multiplex qPCR); however, the number of target genes that can be simultaneously detected in a multiplex qPCR reaction is limited to up to five genes (7). Also, it is technically challenging to design primers/probes and optimize the reaction conditions (e.g., annealing temperature, primer concentrations) to minimize the formation of primer dimers and competition for resources (DNA polymerase, dNTPs) among assays (8, 9).

High-throughput qPCR (HT-qPCR) is another approach to simultaneously detect and quantify multiple target genes. In HT-qPCR, multiple singleplex qPCR assays are simultaneously run in nanoliter-volume chambers (or wells) present in high densities on a chip. Three types of HT-qPCR platforms are commercially available: Biomark (Standard BioTools), OpenArray (Thermo Fisher Scientific), and SmartChip (Takara Bio) systems. These platforms are compatible with both probe and intercalating dye-based assays, although different assay chemistries cannot be used in the same run (6). These HT-qPCR platforms have been used to quantify pathogens (10–14), ARGs (15, 16), nitrogen cycle genes (17, 18), and other genes relevant to the microbiology fields.

The main differences among these HT-qPCR platforms are the number and the volume of qPCR reaction chambers/wells and the method of loading DNA samples and qPCR reagents (6). For example, the Biomark 96.96 Dynamic Array (Standard BioTools) has 9,216 6.7 nL chambers for 96 samples and 96 assays that are loaded and mixed through microfluidic channels (i.e., microfluidic qPCR). OpenArray plate has 3,072 33 nL through-holes, each of which contains assay reagents preloaded by the manufacturer (Thermo Fisher Scientific). DNA samples are loaded to the OpenArray plate using the dispenser (AccuFill system, Thermo Fisher Scientific). SmartChip MyDesign chip has 5,184 100 nL wells, to which DNA samples and assays are loaded using SmartChip NanoDispenser (Takara Bio).

The volume of the reaction chambers can influence the accuracy and sensitivity of the qPCR reaction. To be detected by PCR, at least one copy of the target gene must be present in a chamber. Therefore, the small reaction volume of the HT-qPCR system means a higher detection limit than that of conventional qPCR. For example, to detect one copy in a 6.7 nL reaction chamber of the Biomark 96.96 Dynamic Array, the DNA sample should contain >675 copies/µL (when 1 µL reaction contains 0.227 µL DNA). For reliable quantitative results, it is better to have a target gene at >10^3^–10^4^ copies/µL in the DNA sample (6), which is often too high for environmental and clinical samples (19). Reactions on the SmartChip system are performed in larger-volume chambers (100 nL), and therefore, the detection limit would be lower than that of the Biomark platform. Nonetheless, larger reaction volumes need more reagents and larger volumes of sample DNA, which could increase the overall reagent cost.

Specific target amplification (STA) is a preamplification reaction performed with all primers used for HT-qPCR (i.e., multiplex PCR) with a small number of PCR cycles (e.g., 10–14 cycles). STA has been successfully used to increase the sensitivity of HT-qPCR performed with Biomark (10, 11, 16, 20–22), but its use is less common on the SmartChip platform, with previous applications mostly on eDNA studies of rare and invasive species (23–25). Because SmartChip uses a larger reaction chamber volume than Biomark (100 nL vs 6.7 nL), STA reaction may not be necessary for most applications. However, this has not been experimentally tested.

The objective of this study was to systematically compare the quantitative performance of Biomark HD and SmartChip platforms. Both TaqMan-probe assays (for pathogens and microbial source tracking [MST] markers) (20) and intercalating dye-based assays (for antibiotic resistance genes) (16) were used for the comparison. Wastewater influent and effluent samples (20) and stool samples from patients with heavy antibiotic exposure (26) were used with and without STA reaction for HT-qPCR.

## MATERIALS AND METHODS

### qPCR assays

Previously developed and validated TaqMan-probe assays and intercalating dye-based assays were used in this study. TaqMan-probe assays (*n* = 54) target 22 bacterial and five protozoan pathogens, two fecal indicator bacteria (FIB), and 25 MST marker gene sequences (20). TaqMan probe-based assays are hereafter referred to as MST assays. Intercalating dye-based assays (*n* = 47) target 39 antibiotic and five heavy metal resistance genes and three integrase genes (16). Intercalating dye-based assays are hereafter referred to as ARG assays. The list of target genes and their primer and probe sequences is shown in Tables S1 and S2. These primers were previously verified to cause no major amplification biases during the STA reaction (16, 20).

### Clinical and environmental samples

Stool samples were previously collected from patients with acute myeloid leukemia who received multiple antibiotics over several weeks of hospitalization (26). DNA was extracted using the DNeasy PowerSoil DNA isolation kit (Qiagen). Detailed study design is described in Rashidi et al. (26). The trial protocol and informed consent were approved by the University of Minnesota Institutional Review Board. DNA from 20 representative samples was tested with the ARG assays, and 16 of these samples were also tested with the MST assays. Stool samples tested in this study are completely de-identified.

Domestic wastewater samples were previously collected from 10 wastewater treatment plants located in geographically distinct regions in the USA (20). Biomass in wastewater effluent was concentrated using a dead-end hollow-fiber REXEED 25S ultrafiltration membrane and then backflushed with a sterile backflushing solution. The backflushed effluent samples and influent samples were subsequently used for nucleic acid extraction with the AllPrep PowerViral DNA/RNA Kit (Qiagen). Detailed extraction protocols were previously described by Hill et al. (20). DNA from the influent and effluent samples (*n* = 10 each; a total of 20 samples) was tested with the ARG and MST assays.

### Specific target amplification (STA)

The STA reaction was performed to increase the concentrations of the target gene fragments using the Prelude PreAmp Master Mix (Takara Bio). For the MST assays, a primer mix was prepared by combining 54 different primer pairs to reach a final concentration of 400 nM for each primer. For the ARG targets, a primer mix was prepared by combining 47 different primer pairs to reach a final concentration of 200 nM for each primer. The STA reaction (8 µL) contained 4 µL of 2× Prelude PreAmp Master Mix, 2 µL of DNA template, 1 µL of 400 nM (MST) or 200 nM (ARG) primer mix, and 1 µL of nuclease-free water. Standards were synthesized by combining 54 (MST) and 47 (ARG) gBlock DNA fragments (Integrated DNA Technologies), each containing target gene sequences, and were serially diluted to obtain ranges between 2 × 10^6^ to 2 × 10^0^ copies/µL and 1 × 10^6^ to 1 × 10^0^ copies/µL for MST and ARG assays, respectively. The serially diluted standards and no template controls (NTC; nuclease-free water) were also subjected to the STA reaction and used for the downstream analysis.

**Fig 1:**
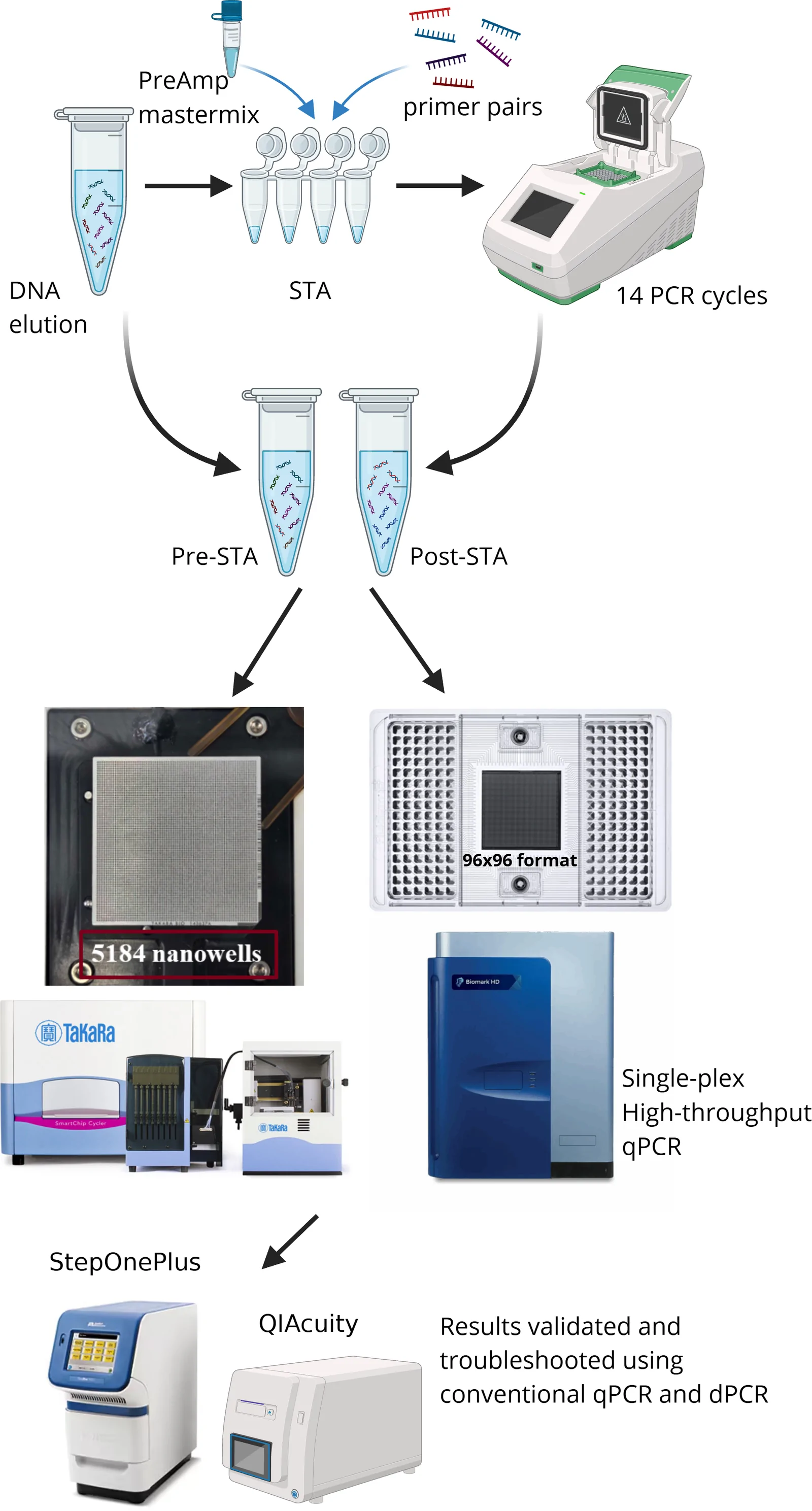
Schematic diagram of sample processing and analysis workflow. Specific target amplification (STA) was used to increase the concentration of target genes prior to high-throughput qPCR (HT-qPCR) analysis. Both pre-STA and post-STA samples were analyzed on the HT-qPCR platforms. For selected assays, conventional qPCR (StepOnePlus; Applied Biosystems) and digital PCR (QIAcuity One; Qiagen) were used for assay validation and troubleshooting.

The STA reaction was run using a Veriti 96-well thermal cycler (Thermo Fisher Scientific) with the following conditions: initial activation at 95°C for 2 min, followed by 14 cycles of 95°C for 10 s and 60°C for 4 min. For intercalating dye-based ARG assays, the STA products were treated with Exonuclease I (New England Biolabs) to remove unused primers and then diluted 5-fold with DNA suspension buffer (10 mM Tris, 0.1 mM EDTA, pH 8.0) for downstream analysis. For TaqMan probe-based MST assays, the STA products were diluted 5-fold with DNA suspension buffer without the Exonuclease treatment and used for downstream analysis. The sample processing schematic is shown in Fig. 1.

### HT-qPCR with Biomark HD system

Both the undiluted DNA samples (i.e., pre-STA samples) and STA-amplified, 5-fold-diluted STA products (i.e., post-STA samples) were tested with HT-qPCR, along with the pre-STA and post-STA standards, using the Biomark HD Real-Time PCR system (Standard BioTools). The intercalating dye-based ARG assays (ARG chip) and the TaqMan probe-based MST assays (MST chip) were run separately using 96.96 Dynamic Array IFC chips (Standard BioTools) following the manufacturer’s protocol and procedures previously described (16, 20). Briefly, for the MST chip, the assay mix (5 µL) containing 1× Assay Loading Reagent (Standard BioTools), 2 μM primer pair, and 1 μM probe, and the sample mix (5 µL) containing 1× SsoAdvanced Universal Probe Supermix (Bio-Rad), 1× GE Sample Loading Reagent (Standard BioTools), and 2.25 μL of pre- or post-STA sample were loaded into the assay and sample wells, respectively, of the IFC chip. For the ARG chip, the assay mix (5 µL) containing 1× Assay Loading Reagent (Standard BioTools) and 5 μM primer pair, and the sample mix (5 µL) containing 1× SsoFast EvaGreen Supermix with low ROX (Bio-Rad), 1× DNA Binding Dye (Standard BioTools), and 2.25 μL of pre- or post-STA sample were loaded into the assay and sample wells, respectively, of the IFC chip. After loading the assay mix and sample mix onto the IFC chip, the IFC Controller MX (Standard BioTools) was used to mix the assay and sample solutions. This resulted in the total number of reactions run on each IFC chip being 9,216, with a final volume of 6.7 nL for each reaction. HT-qPCR was performed using the conditions previously described by Hill et al. (20) for the MST chip and Sandberg et al. (16) for the ARG chip. Briefly, for the MST chip, thermal mixing was performed at 70°C for 40 min, followed by 60°C for 30 s. Cycling was performed with an initial activation at 98°C for 3 min, followed by 40 cycles of 98°C for 10 s and 60°C for 30 s. For the ARG chip, thermal mixing was performed at 70°C for 40 min, followed by 60°C for 30 s. Cycling was performed with an initial activation at 95°C for 1 min, followed by 40 cycles of 96°C for 5 s and 60°C for 20 s. Additionally, melt curve analysis was performed from 60°C to 95°C with a heating rate of 1°C every 3 s.

### HT-qPCR with SmartChip system

The same pre- and post-STA samples described above were tested for HT-qPCR with the SmartChip Real-Time PCR System (Takara Bio). Similar to the Biomark, the ARG assays and the MST assays were run separately using the SmartChip MyDesign Kit (Takara Bio). The SmartChip TB Green Gene Expression Master Mix (Takara Bio) and the SmartChip Probe qPCR Master Mix (Takara Bio) were used for the ARG chip and the MST chip, respectively. The DNA samples, primers, and probes (only for the MST assays) were combined with the Master Mix described above and dispensed into the NanoWells of the SmartChip MyDesign Chip. The reaction volume of each NanoWell of the MyDesign Chip is 100 nL. The final primer concentration for the ARG assays was 500 nM, whereas the final primer and probe concentrations were 200 nM and 100 nM, respectively, for the MST assays (16, 20).

After loading the MyDesign chip, the SmartChip Real-Time PCR Cycler (Takara Bio) was used for thermal cycling with the following conditions for the MST chip: initial activation at 95°C for 9 min and 45 s, followed by 40 cycles of 95°C for 10 s and 60°C for 53 s. For the ARG chip, the following conditions were used: initial activation at 95°C for 3 min, followed by 40 cycles of 95°C for 30 s and 60°C for 30 s. Additionally, a melt curve analysis was performed from 60°C to 97°C with a gradient of 0.4°C per step.

### Conventional qPCR

In cases where differences were observed between the results from the Biomark HD platform and the SmartChip platform, conventional qPCR was performed to compare the results and determine which platform produced results more similar to those obtained by conventional qPCR. Three MST assays (BacCanUCD, Cperf, and *ssrA*) and four ARG assays (*aadA5*, *ermF*, *mexB*, and *tet(X*)) were selected for comparison using post-STA samples to enable cross-platform evaluation.

Conventional qPCR was performed using a StepOnePlus Real-Time PCR system (Applied Biosystems). For the MST assays, Premix *Ex Taq* Master Mix, Rox Plus (Takara Bio), was used with final primer and probe concentrations of 400 nM and 200 nM, respectively. The thermal cycling conditions were as follows: initial activation at 95°C for 20 s, followed by 40 cycles of 95°C for 1 s and 60°C for 20 s.

For the ARG assays, SsoFast EvaGreen Supermix (Bio-Rad) was used with a final primer concentration of 500 nM. The thermal cycling conditions were as follows: initial activation at 95°C for 3 min, followed by 40 cycles of 98°C for 5 s and 60°C for 30 s. A melt curve analysis was performed from 60°C to 95°C with a gradient of 0.3°C per step.

### Digital PCR

Digital PCR (dPCR) was performed using the QIAcuity One Digital PCR System (Qiagen) with the QIAcuity Nanoplate 8.5k 96-well plate (Qiagen) to evaluate the assay’s amplification performance and the presence of PCR inhibitors in the pre-STA DNA samples. The seven assays selected for conventional qPCR were individually used in singleplex dPCR. For the MST assays, a 15 µL reaction mix containing 1× QIAcuity Probe PCR Master Mix (Qiagen), 800 nM primers, 400 nM probe, and 1 µL of DNA sample was used. For the ARG assays, a 15 µL reaction mix containing 1× QIAcuity EG PCR Master Mix (Qiagen), 400 nM primers, and 1 µL of DNA sample was used. After vortexing and brief centrifugation of the reaction mixtures prepared in standard 96-well plates, 12 µL of each reaction mixture was transferred to a QIAcuity 8.5K 96-well Nanoplate (Qiagen). The following cycling conditions were used for both the MST and ARG plates: initial activation at 95°C for 2 min, followed by 40 cycles of 95°C for 15 s and 60°C for 30 s. An additional cooling step at 40°C for 5 min was included for the ARG plates before imaging. Imaging and data analysis were performed using the QIAcuity Software Suite v3.1.0.0 with the default settings.

### Cost analysis

To assess the cost of HT-qPCR runs on the two platforms and compare them with conventional qPCR, reagent and consumable costs were obtained from the manufacturers and reflected the listed prices as of June 2026. The cost per reaction (i.e., sample–assay combination) was calculated by prorating the costs of reagents (e.g., master mix and assays) and consumables (e.g., chips) on a per-reaction basis. The cost comparison was based on a 96.96 Dynamic Array IFC configuration for the Biomark HD platform and a 54-assay × 96-sample configuration for the SmartChip platform. These estimates do not include labor, common laboratory consumables (e.g., 96-well plates, plate seals, and pipette tips), costs associated with the pre-amplification reaction, capital costs of the instruments, or costs associated with instrument maintenance and service contracts for the HT-qPCR systems.

### Statistical analysis

To assess detection agreement between pre- and post-STA samples and between the two HT-qPCR platforms, a positivity threshold was applied to facilitate comparison and minimize the influence of false-positive detections. Because most assays have detection limits of 10¹–10² copies/µL and the likelihood of non-specific amplification (i.e., false-positive signals) increases at high quantification cycle (Cq) values (27), only samples with concentrations exceeding 10² copies/µL were considered positive for the detection agreement analyses. In addition, mixed-effects logistic regression was used to evaluate differences in binary detection outcomes between the two HT-qPCR platforms, with platform included as a fixed effect and assay and sample included as random effects. The regression model was fitted using the lme4 package (version 2.0.1) (28) in R version 4.4.1 (29).

For quantitative agreement analyses, only samples detected both before and after STA or on both HT-qPCR platforms were included. Lin’s concordance correlation coefficient (CCC) was calculated using the DescTools package version 0.99.60 (30) in R version 4.4.1 using RStudio version 2023.6.0.421 (31). In addition, CCC values were also calculated separately for individual assays with more than 10 paired detections. Linear regression analyses were also performed using the built-in stats package, and coefficients of determination (*R*²) were calculated from the fitted models. Regression lines and corresponding 95% confidence intervals were generated and visualized using the ggplot2 package version 3.5.2 (32).

To evaluate the impact of primer melting temperature (Tm) on quantitative agreement between pre- and post-STA measurements for ARG assays, CCC values were plotted against the average Tm of each primer pair. Primer Tm values were estimated using the IDT PrimerQuest Tool, and the resulting plots were generated using Origin 2026 (version 10.3.0.197).

## RESULTS

### Assay performance

Two MST assays (AllBac and BacGen) targeting *Bacteroides* spp. were excluded because most samples exceeded the upper detection limit of the highest standard concentration (2 × 10^6^ copies/µL). One heavy metal resistance gene assay (*nikA*) was excluded from the ARG assays due to lack of amplification in the standard curves on both HT-qPCR platforms. Two clinical samples were excluded from the analysis of STA effects on ARG assays due to insufficient pre-STA sample volume.

**Fig 2:**
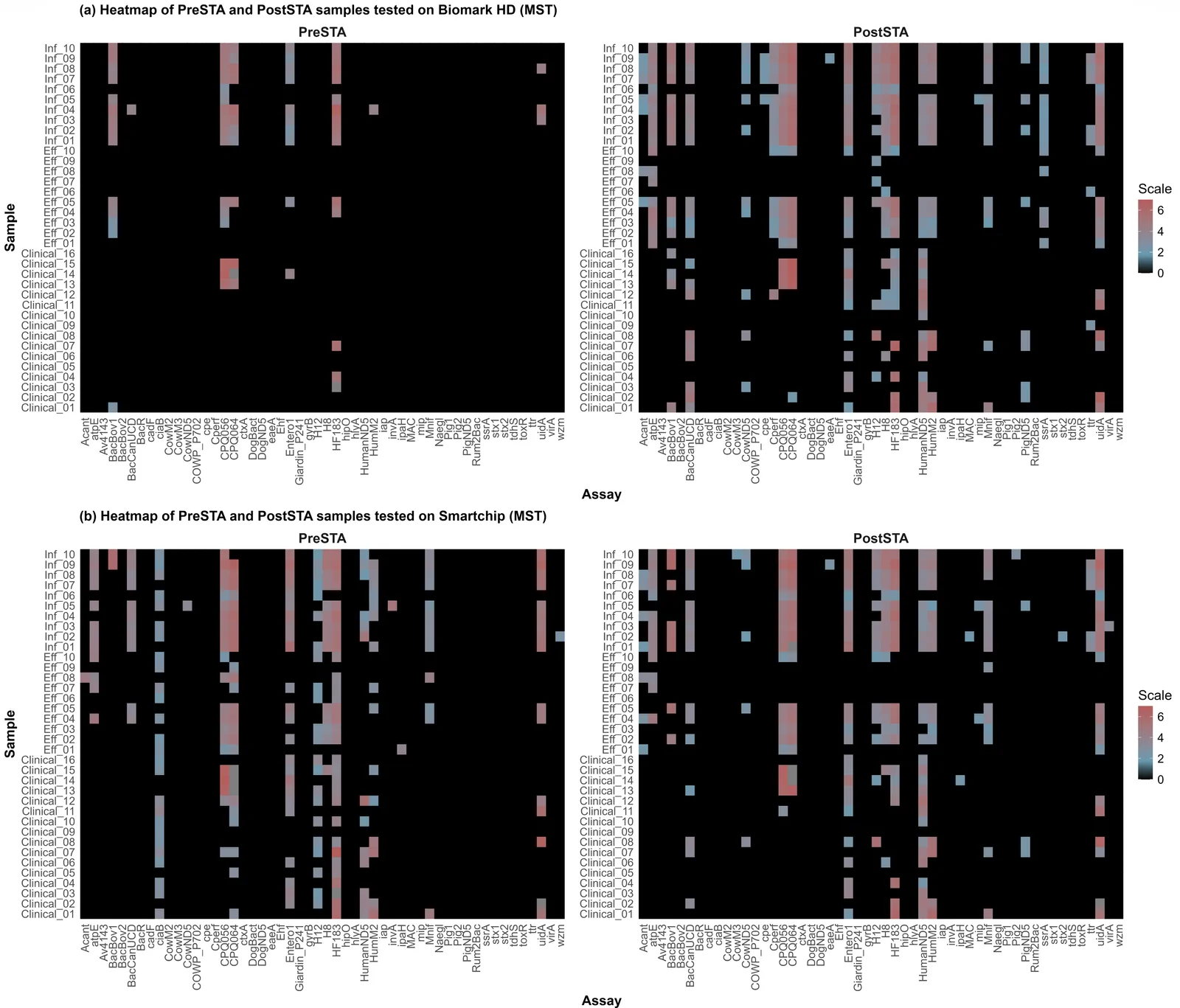
Heatmaps showing the detection of microbial source tracking (MST) markers, fecal indicator bacteria, and pathogens in samples before and after specific target amplification (STA) analyzed on the (**a**) Biomark HD platform (Standard BioTools) and (**b**) SmartChip platform (Takara Bio). Only positive detections (>10^2^ copies/µL) were included in the heatmap. The scale represents log_10_(copies)/µL. STA had less impact on detection frequency and target quantification on the SmartChip platform.

For the MST assays, the STA reaction increased detection frequency on the Biomark HD platform. Without STA, positive detection (>10^2^ copies/µL) was seen only for eight assays. The number of detections was 71 out of 1,872 sample × assay combinations, whereas it increased to 330 (for 22 assays) with STA (Fig. 2). In contrast, the impact of STA on the SmartChip platform was minimal. Without STA, the number of detections (>10^2^ copies/µL) was 252 (for 19 assays) out of 1,872 sample × assay combinations. The number of detections was similar even with STA (262 detections; 25 assays).

A similar pattern was observed for the intercalating dye-based ARG assays. Without the STA process, the number of detections (>10² copies/µL) on the Biomark HD platform was 31 out of 1,748 sample × assay combinations. With STA, the number of detections (>10² copies/µL) increased to 635 out of 1,748 sample × assay combinations on the Biomark HD platform. In contrast, the number of detections (>10² copies/µL) on the SmartChip platform was similar before and after STA, with 773/1,748 detections (for 45 assays) and 748/1,748 detections (for 43 assays), respectively.

### Effect of specific target amplification

**Fig 3:**
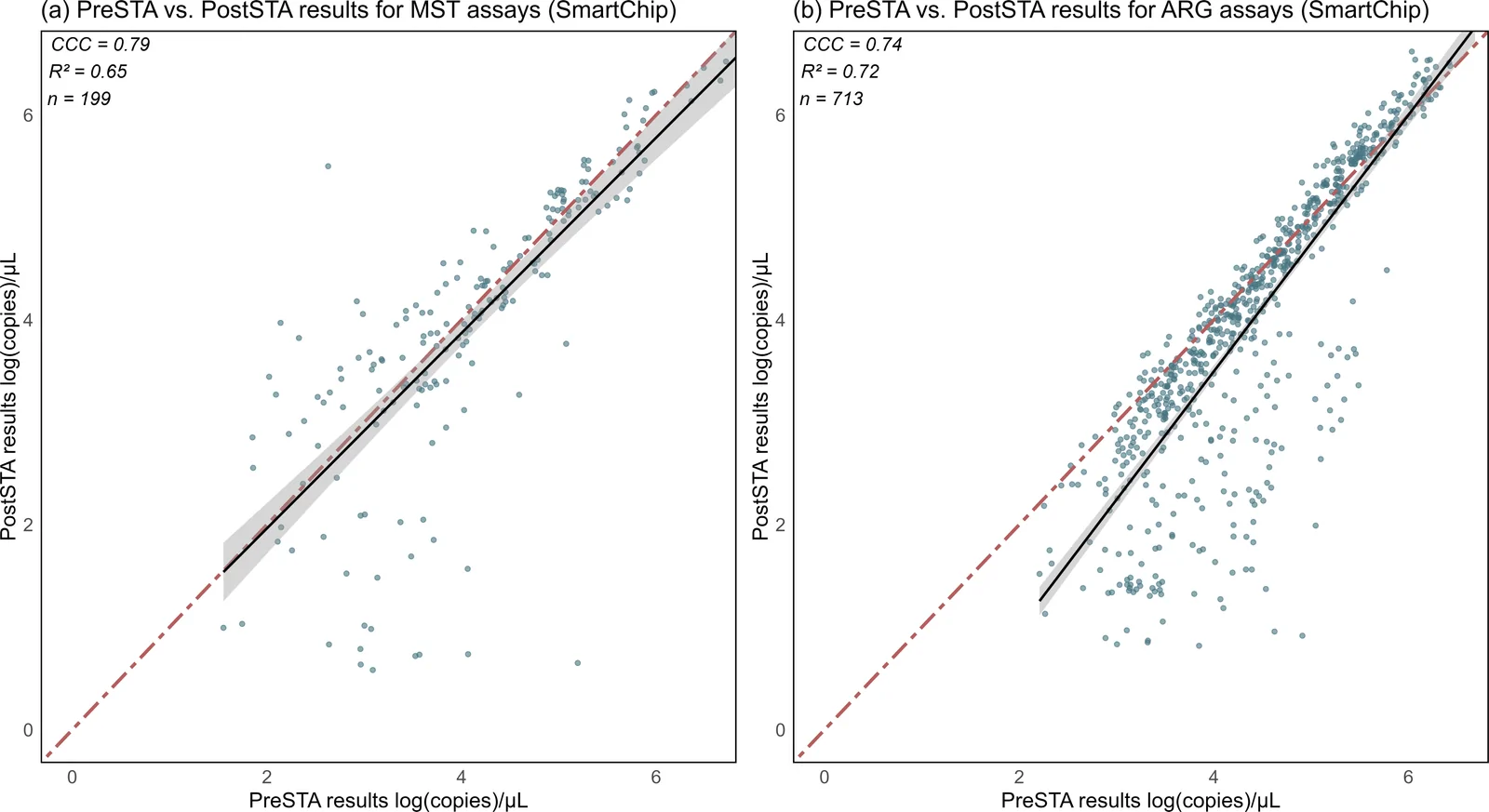
Correlation plots comparing quantitative results before and after specific target amplification (STA) for (**a**) microbial source tracking (MST) and (**b**) antibiotic resistance gene (ARG) assays analyzed on the SmartChip platform (Takara Bio). The red dashed line indicates perfect agreement (y = x), the black line represents the linear regression fit, and the gray shaded area represents the 95% confidence interval. Coefficients of determination (*R*²) for the fitted linear regression models, Lin’s concordance correlation coefficients (CCC), and the number of samples detected are shown.

Because the SmartChip platform detected many positive sample × assay combinations both before and after STA for the ARG and MST assays, we further evaluated the effect of STA on detection frequency and quantification. Detection agreement was analyzed by comparing samples before and after STA. For the 1,872 sample × assay combinations of the MST assays, 91.3% (*n* = 1,710) showed the same binary results (i.e., detected or not detected using 10² copies/µL as the cutoff) before and after STA. Similarly, detection frequency was comparable between pre- and post-STA samples for the ARG assays, with 87.4% (*n* = 1,527) of the 1,748 sample × assay combinations showing the same binary results before and after STA. For samples that were detected both before and after STA, correlation analysis showed similar quantitative agreement for both the MST and ARG assays, with CCC values of 0.79 and 0.74, respectively (Fig. 3). In addition, correlation analyses were performed individually for assays that produced more than 10 detections in both pre- and post-STA samples. Of the MST assays (*n* = 12) meeting this criterion, one assay (H12) exhibited low concordance, with CCC and *R*² values of 0.06 and 0.01, respectively (Fig. S1). Of the ARG assays (*n* = 37) meeting this criterion, some assays, such as *bla*_SHV_, *mexB*, and *sul3*, showed low CCC values (<0.4) and had negative intercepts in the fitted correlations. This suggests that the gene concentrations measured with these assays after the STA process were underestimated. Interestingly, all of these assays had higher primer Tm than the annealing temperature of the reaction (60°C) (Fig. S2).

### Differences across the HT-qPCR platforms

**Fig 4:**
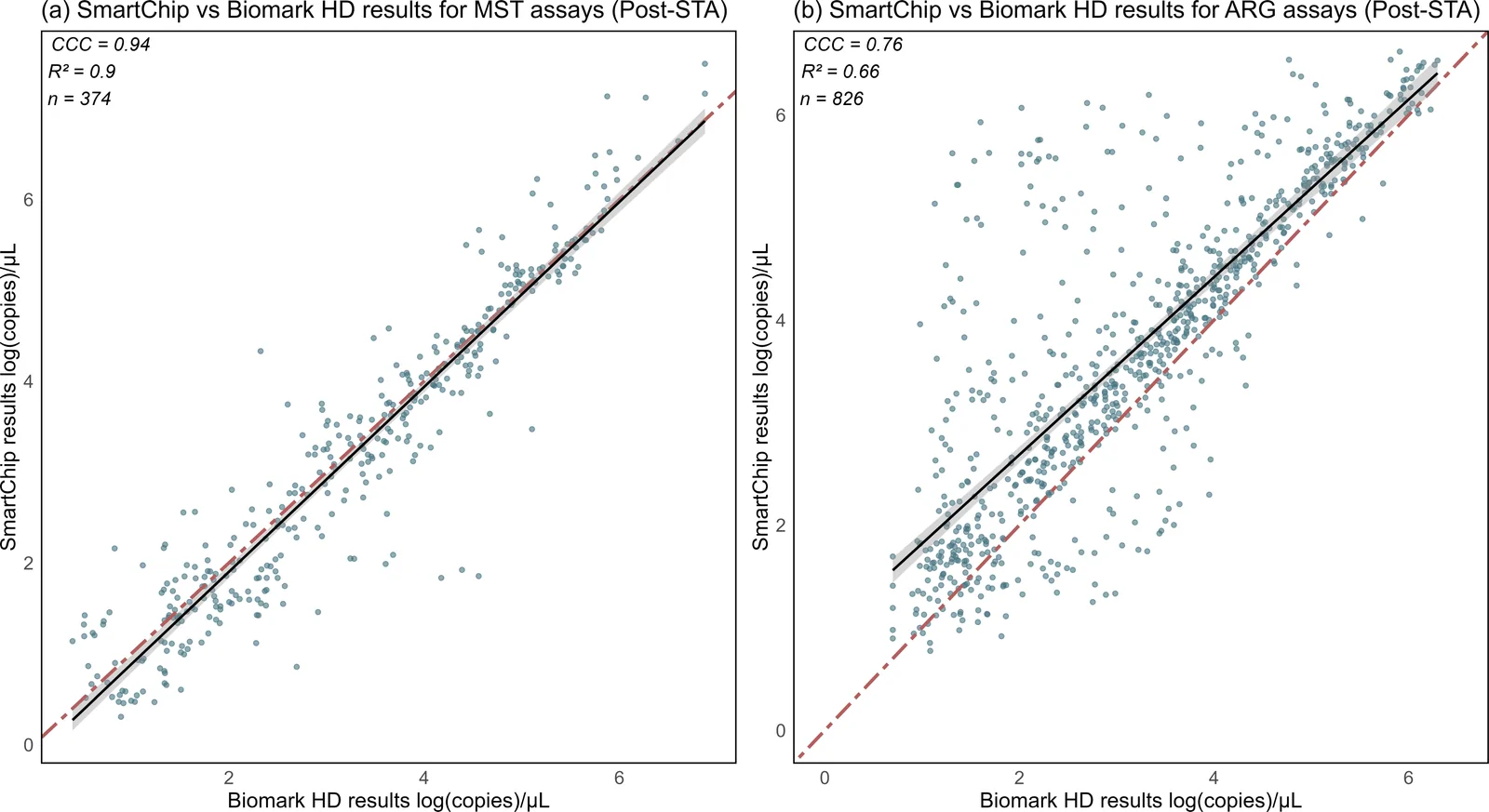
Correlation plots comparing quantitative results produced by the SmartChip (Takara Bio) and Biomark HD (Standard BioTools) platforms for (**a**) microbial source tracking (MST) and (**b**) antibiotic resistance gene (ARG) assays. The red dashed line indicates perfect agreement (y = x), the black line represents the linear regression fit, and the gray shaded area represents the 95% confidence interval. Coefficients of determination (*R*²) for the fitted linear regression models, Lin’s concordance correlation coefficients (CCC), and the number of samples detected are shown.

Given that STA significantly increased detection on the Biomark HD platform, cross-platform comparisons between the Biomark HD and SmartChip were performed only using post-STA samples. Overall, target gene concentrations measured using the two platforms were comparable (Fig. 4), with CCC values of 0.94 and 0.76 for MST and ARG assays, respectively. Of the 14 MST assays and 39 ARG assays that produced more than 10 detections on both platforms, MST assays showed a higher degree of quantitative agreement between the two platforms (Fig. S3).

In addition to quantitative comparisons, detection agreement was analyzed between the two platforms. For the MST assays, 94.3% (*n* = 1,766) of 1,872 assay-sample combinations showed the same binary results (i.e., detected or not detected when using 10^2^ copies/µL as the cutoff) on the two platforms. Of the 106 discordant reactions (5.7%), 87 and 19 were detected (>10² copies/µL) only on the Biomark HD and SmartChip platforms, respectively (Fig. S4a). Mixed-effects logistic regression showed that, when using 1 × 10^2^ copies/µL as the detection cutoff, the SmartChip platform exhibited significantly lower odds of detection than the BioMark HD platform after accounting for variability among assays and samples (*P* < 0.001). When examining individual assays, two MST assays (Cperf and *ssrA*) showed more than 10 inconsistent results between the platforms. On Biomark HD, 15 and 14 samples showed positives for Cperf and *ssrA* assays, respectively, whereas no samples were detected on SmartChip. In addition, no standards produced Cq values for Cperf and *ssrA* on the SmartChip platform.

For the ARG assays, the same binary results were obtained for 87.4% (*n* = 1,609) of the 1,840 assay–sample combinations between the two platforms. Of the 231 discordant reactions (12.6%), 60 and 171 were detected (>10² copies/µL) only on the Biomark HD and SmartChip platforms, respectively (Fig. S4b). Mixed-effects logistic regression showed that the SmartChip platform had higher odds of detection for the ARG assays than the BioMark HD platform, after accounting for variability among assays and samples and using 1 × 10² copies/µL as the detection threshold (*P* < 0.001).

### Comparison with conventional qPCR

Because Cperf and *ssrA* were detected only on the Biomark HD platform, we ran these assays using conventional qPCR to determine which platform (Biomark HD vs SmartChip) provided results more similar to those from qPCR. In addition, the BacCanUCD assay was also selected for conventional qPCR because it showed the lowest CCC and *R*^2^ values (0.41 and 0.33, respectively) between the Biomark HD and SmartChip results (Fig. S3a).

**Fig 5:**
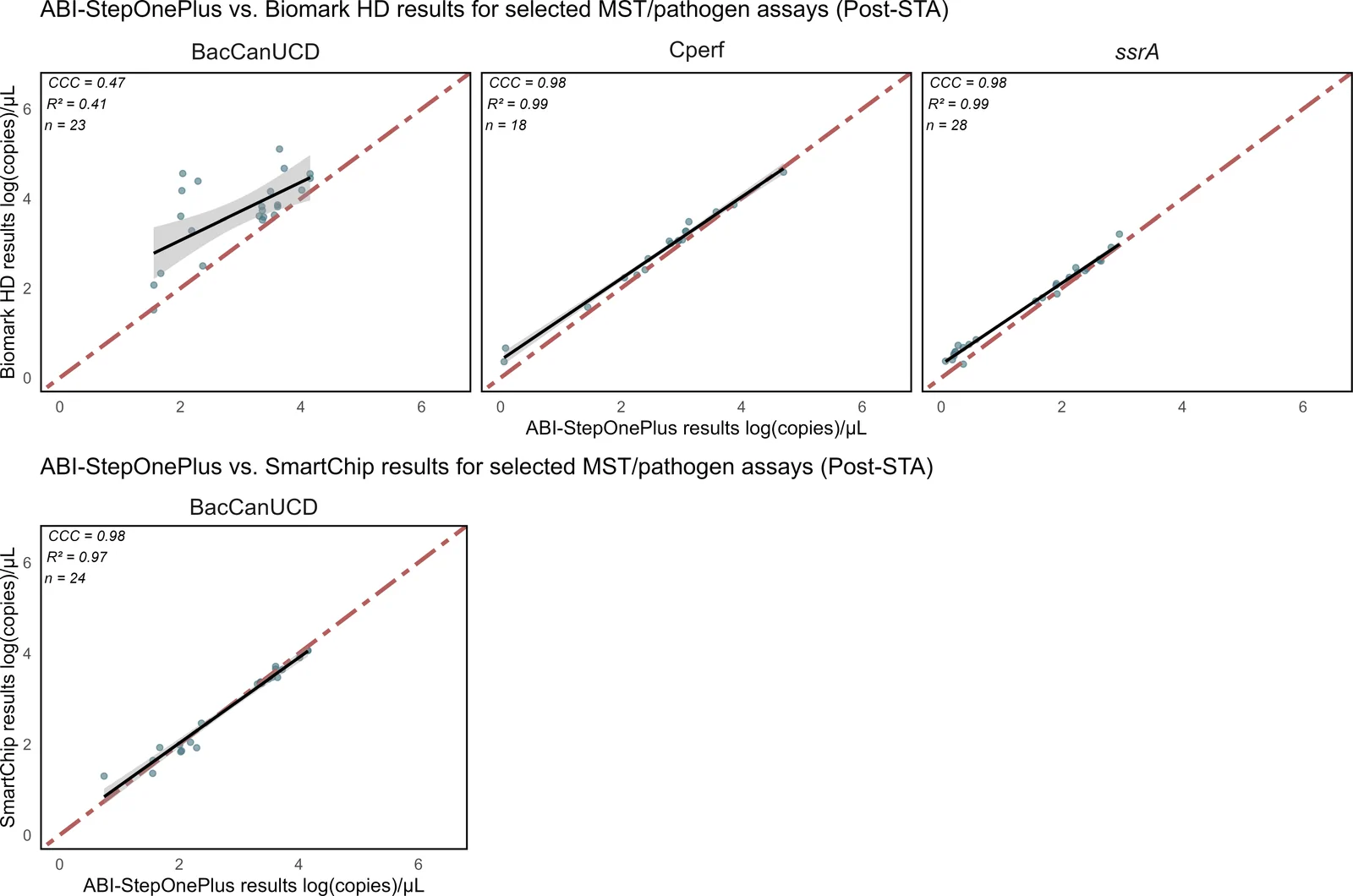
Correlation plots of selected microbial source tracking (MST) assays comparing results obtained using the StepOnePlus (Applied Biosystems) and the (top) Biomark HD (Standard BioTools) or (bottom) SmartChip (Takara Bio) platforms. The red dashed line indicates perfect agreement (y = x), the black line represents the linear regression fit, and the gray shaded area represents the 95% confidence interval. Coefficients of determination (*R*²) for the fitted linear regression models, Lin’s concordance correlation coefficients (CCC), and the number of samples detected are shown for each assay. No Cq values were generated for the Cperf and *ssrA* assays on the SmartChip platform, whereas the Biomark HD results were similar to those obtained with the StepOnePlus.

The occurrence of Cperf and *ssrA* was verified by conventional qPCR, with good agreement with the results obtained from the Biomark HD platform (CCC = 0.98 and *R*² = 0.99 for both assays; Fig. 5). However, for BacCanUCD, where differences were observed between the Biomark HD and SmartChip platforms, results from the SmartChip platform were more consistent with conventional qPCR (CCC = 0.98 and *R*² = 0.97; Fig. 5).

Conventional qPCR was also used to examine the inconsistent ARG assay results observed between pre-STA and post-STA samples on the SmartChip platform as well as between platforms for post-STA samples. Four ARG assays were selected for conventional qPCR, including *mexB*, which showed a low CCC value between pre-STA and post-STA samples (0.22; Fig. S1b) and between platforms (0.41; Fig. S3b), *ermF* and *tet(X*), which showed good agreement between pre-STA and post-STA samples (CCC ≥0.95; Fig. S1b) but poor agreement between platforms (CCC = 0.01 and 0.06, respectively; Fig. S3b), and *aadA5*, which showed good agreement both between pre- and post-STA samples and between platforms.

**Fig 6:**
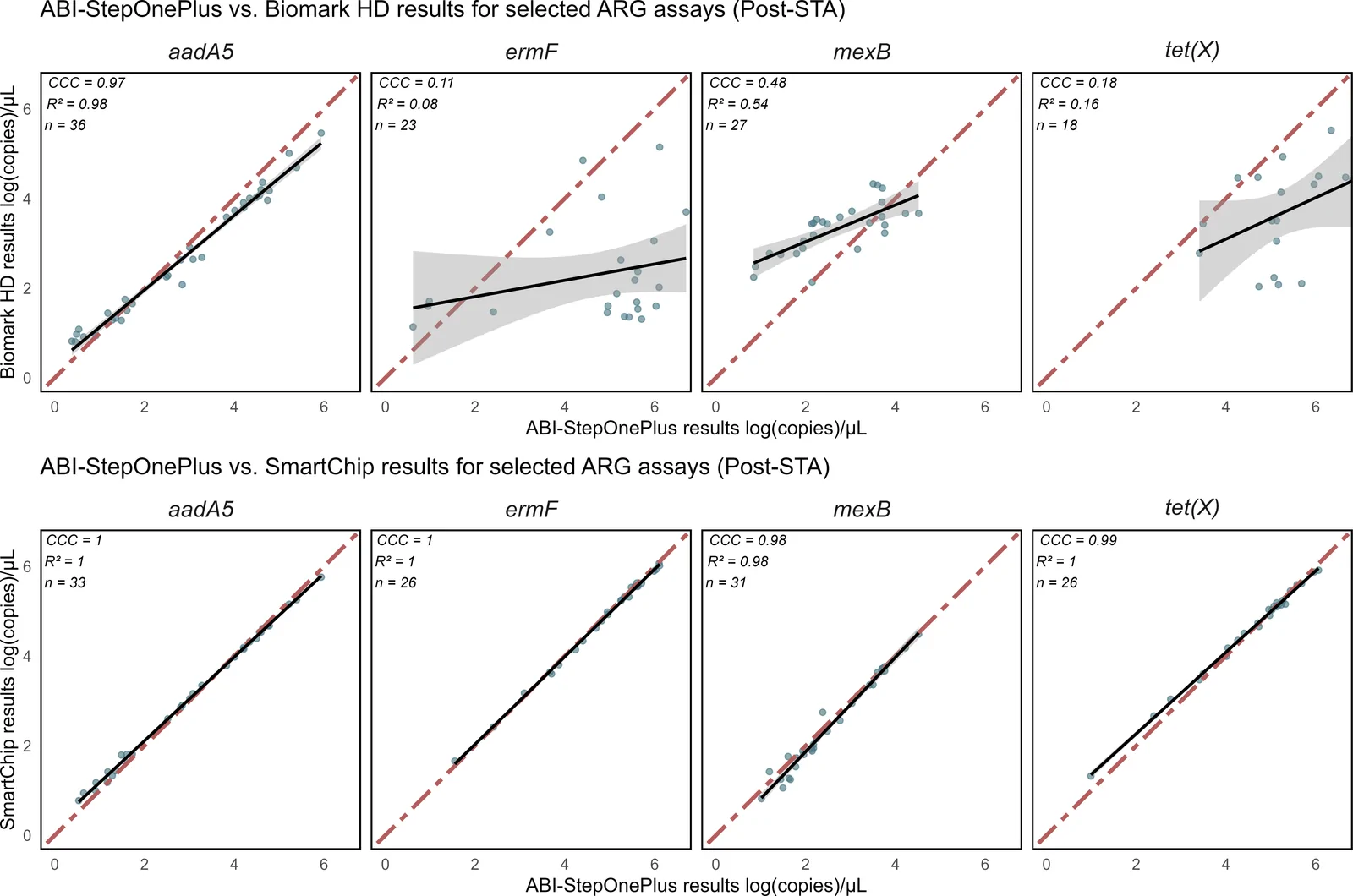
Correlation plots of selected antibiotic resistance gene (ARG) assays comparing results obtained using the StepOnePlus (Applied Biosystems) and the (top) Biomark HD (Standard BioTools) or (bottom) SmartChip (Takara Bio) platforms. The red dashed line indicates perfect agreement (y = x), the black line represents the linear regression fit, and the gray shaded area represents the 95% confidence interval. Coefficients of determination (*R*²) for the fitted linear regression models, Lin’s concordance correlation coefficients (CCC), and the number of samples detected are shown for each assay. For the selected assays, SmartChip results were more similar to those obtained with the StepOnePlus.

For the *aadA5* assay, conventional qPCR provided similar results to those from the two HT-qPCR platforms (CCC = 0.97 [Biomark HD] and 1 [SmartChip], *R*² = 0.98 [Biomark HD] and 1 [SmartChip]; Fig. 6), confirming the detections and strong agreement between platforms. In cases where discrepancies were observed between the Biomark HD and SmartChip platforms (e.g., *ermF* and *tet(X*)), SmartChip results were more similar to those obtained by conventional qPCR, with both CCC and *R*² values ≥ 0.99 (Fig. 6). For assays showing differences between pre-STA and post-STA samples (e.g., *mexB*), post-STA SmartChip results remained strongly aligned with conventional qPCR done with post-STA samples (CCC = 0.98 and *R*² = 0.98; Fig. 6), suggesting that observed discrepancies between pre- and post-STA results may be attributable to the amplification bias introduced during the STA process.

### Digital PCR

Endpoint fluorescence measurements from individual partitions on the QIAcuity nanoplate (Qiagen) provided additional insight into the performance of the selected assays. Similar to conventional qPCR, dPCR results confirmed the detection of BacCanUCD, Cperf, and *ssrA* in the pre-STA samples. Although all four selected ARG assays were successfully detected by dPCR, many *mexB* partitions exhibited fluorescence signals within the intermediate region between the positive and negative clusters, a pattern commonly referred to as “rain” (Fig. S5) (33) that is often associated with suboptimal amplification efficiency.

The dPCR results also show differences in the distribution of fluorescence intensity between wastewater and fecal samples (Fig. S6). The decreased signal-to-noise ratio observed in wastewater samples is likely due to the presence of PCR inhibitors. Interestingly, this pattern was observed only for wastewater samples analyzed with the *tet(X*) assay, suggesting that PCR inhibitors may affect different assays to varying degrees.

### Cost comparison of HT-qPCR platforms and conventional qPCR

Overall, when comparing the 54-assay × 96-sample configuration on the SmartChip platform with the 96.96 Dynamic Array IFC configuration on the Biomark HD platform, the SmartChip system had a lower per-run cost ($901 and $1,883 for the SmartChip and Biomark HD platforms, respectively; Table S3). However, the prorated cost per reaction was relatively similar between the two platforms ($0.16 and $0.21 per reaction for the SmartChip and Biomark HD, respectively). While conventional qPCR typically costs approximately $0.50–$1.50 per reaction, depending on the assay (34), HT-qPCR provides some cost benefits compared with conventional qPCR. However, it is important to note that the prorated cost per reaction for HT-qPCR is based on a full chip run, with all wells being used for sample testing.

## DISCUSSION

Since the launch of HT-qPCR platforms such as the Biomark HD and SmartChip Real-Time PCR systems in the early 2010s, they have gained increasing popularity across multiple fields of study, including environmental pathogen and ARG monitoring (10, 11, 20, 35, 36), functional gene detection (37, 38), and various clinical studies (39–41). In this study, we evaluated the performance of the two widely used HT-qPCR platforms and assessed the necessity of target pre-amplification (i.e., STA) prior to HT-qPCR runs. While STA was necessary to detect most of the genes by the Biomark HD platform, it was not necessary, at least for our wastewater and fecal samples tested, to detect MST marker genes and ARGs by the SmartChip platform. This difference is likely due to the different reaction volume in the HT-qPCR chips (6.7 nL in Biomark HD vs 100 nL in SmartChip) (17, 20).

For the MST assays analyzed in this study, in addition to pathogen and human-associated marker targets, the panel included animal-associated markers (e.g., dog, cow, and pig) that are not expected to be present in clinical samples or at high abundance in domestic wastewater. For the ARG assays, wastewater samples were expected to have a higher detection frequency and abundance of ARGs compared with the fecal samples. For the samples and assays analyzed in this study, detection frequency and target concentrations were similar between pre- and post-STA samples with CCC values of >0.7 for both MST and ARG assays (Fig. 3), suggesting that STA did not negatively influence the quantitative results for these assays. However, some assays, especially those with a higher primer Tm than the annealing temperature, showed low CCC values (<0.4, Fig. S2), with lower concentrations in post-STA than in pre-STA samples. The use of a lower annealing temperature than the primer Tm can increase non-specific binding of primers to the template, the formation of primer dimers, and the likelihood of non-specific product formation (42, 43). This would result in competition between target and non-target amplification, thereby lowering amplification efficiency of the target gene fragments (44). Non-target amplification can occur more frequently in complex environmental or fecal samples with a large amount of background DNA than in simple gBlock or plasmid solutions (27, 45), leading to different amplification efficiencies between standards and environmental/fecal samples. This may explain, at least in part, why STA introduced underestimation of the gene concentrations for assays that had higher Tm than the annealing temperature. For example, when the true concentration of a gene in an environmental sample is 500 copies/µL, the Cq to detect that level can be 28.6 when amplification efficiency is 80%. However, Cq of 28.6 for a standard curve with amplification efficiency of 100% can mean 24.6 copies/µL, significantly underestimating the true concentration (see Fig. S7 for more details). Therefore, for unbiased STA and accurate quantification, it is important to minimize the formation of non-specific product by carefully selecting primers. Based on our results, we recommend using primers that have the Tm within 2°C of the annealing temperature for the STA reaction to minimize potential amplification biases. This study also demonstrates the benefits of evaluating the effects of STA by comparing pre- and post-STA results from a subset of samples when testing large-scale, complex sample sets. Previous studies evaluated the effects of STA by comparing the standard curves generated with and without STA (10, 16, 20, 21); however, because standard curves were generated using short DNA fragments (e.g., gBlock or plasmids), their approach cannot fully capture the occurrence of non-specific binding of primers.

The two HT-qPCR platforms provided comparable quantitative results, although the quantitative agreements were larger for the TaqMan probe-based MST assays than the intercalating dye-based ARG assays (CCC = 0.94 and 0.76, respectively; Fig. 4). This is expected, as TaqMan probe-based assays generally have higher sensitivity and specificity than dye-based assays (46, 47). Interestingly, for the four ARG assays selected for conventional qPCR validation, results from the SmartChip platform were highly correlated with those from the ABI StepOnePlus (average CCC >0.99; Fig. 6). In contrast, substantial discrepancies were observed between the Biomark HD and ABI StepOnePlus platforms for the *ermF*, *mexB*, and *tet(X*) assays (average CCC = 0.26; Fig. 6). One possible explanation for these discrepancies is the smaller reaction volume of the Biomark HD platform (6.7 nL) than the SmartChip (100 nL) and StepOnePlus platforms (10 µL), which may increase variability arising from stochastic partitioning and amplification (48). This effect may be more pronounced for intercalating dye-based assays, which are more susceptible to non-specific binding and PCR artifacts, such as primer dimers, than TaqMan probe-based assays (46, 47). Additionally, the Biomark HD platform uses a lower sample-to-reaction mix ratio (1:4.4) than both SmartChip and conventional qPCR (1:10), which may further contribute to differences in assay performance.

The lower sample-to-reaction mix ratio of the Biomark HD platform could also result in a stronger effect of PCR inhibitors. Environmental and clinical samples contain variable levels of PCR inhibitors (4, 49), as also suggested by our dPCR results for *tet(X*). In our study, samples used for the quantitative agreement analysis are not necessarily the same across assays, as only samples showing positives in both platforms were used for the correlation analysis. Therefore, the occurrence and the levels of PCR inhibitors in the samples used for the agreement analysis could vary by assay. In addition to variability in inhibitor levels among samples, susceptibility to inhibition can vary by assay (50) and can differentially affect target templates within the same sample (51). Our dPCR results also indicate that some assays, such as *aadA5* and *ermF*, are less affected by inhibitors than *tet(X)* (Fig. S6).

Two MST assays, Cperf and *ssrA*, did not produce any amplicons on the SmartChip platform, including gBlock standards. This potentially contributed to the lower odds of detection for the MST assays on the SmartChip platform, as suggested by the mixed-effects logistic regression model. In this study, primer and probe concentrations of 200 nM and 100 nM, respectively, were used based on prior experiments on the Biomark HD platform (20); however, these concentrations might be too low to be detected on the SmartChip platform. We later determined that increasing the primer and probe concentrations of Cperf and ssrA assays to 400 nM and 200 nM, respectively, resulted in successful amplification (data not shown). Both the Biomark HD and SmartChip platforms allow customization of primer and probe concentrations for individual assays, and such optimization may be necessary for accurate quantification (52).

As new MST markers continue to be developed (e.g., pBI143; [53]) and novel ARGs are identified, HT-qPCR has gained increasing popularity across multiple fields, with target selection varying widely depending on research and diagnostic goals. The HT-qPCR can also be applied to the other areas of study, such as N cycling (18) and methane production (38). The results from this study provide a general framework for assay selection in HT-qPCR and can be used alongside the MIQE guidelines 4,54for quantitative PCR (4,54).

1. **DNA/RNA quality and target concentration:** Given that the reaction volumes of HT-qPCR systems are typically on the nanoliter scale and that highly multiplexed preamplification is sometimes required, DNA and RNA samples should be of high quality, with no or minimal presence of PCR inhibitors. DNA/RNA extraction and purification protocols should be established, depending on the sample type, to improve DNA or RNA recovery and reduce inhibitors. Sample process control (e.g., spike-and-recovery approach; [4, 54]) is useful to assess DNA recovery efficiency. We recommend running conventional qPCR targeting the 16S rRNA gene to detect any sign of PCR inhibition (e.g., gentler slope compared to DNA standards) and/or using a UV-Vis spectrophotometer to assess the purity of DNA/RNA elution. Applying amplification efficiency corrections may further improve the accuracy of quantitative results and data reporting (49, 55).
  Additionally, in this study, the concentrations of *Bacteroides* spp. measured by the AllBac and BacGen assays exceeded the highest standard concentration (2 × 10⁶ copies/µL). While high concentrations of *Bacteroides* spp. were expected given that fecal and municipal wastewater samples were analyzed in this study, these results suggest that the range of standards should be adjusted for individual assays based on the expected target concentrations. For example, 2 × 10⁶ copies/µL as the highest standard concentration for *Bacteroides* spp. may be sufficient for surface water samples but not for wastewater samples. It is important to note that, while sample dilution is commonly used to quantify high-abundance targets (e.g., *Bacteroides* spp.), the same sample is used to quantify multiple targets in HT-qPCR runs. Therefore, sample dilution could reduce the chance of detecting low-abundance targets such as pathogens.
2. **Pre-amplification:** Pre-amplification (STA) may be necessary depending on the sample, assay, and the HT-qPCR platform. In this study, STA was not required for SmartChip analysis of fecal and wastewater samples, but it may be needed for other sample types (e.g., surface water samples) and for rare targets. The need for STA should be examined by comparing pre- and post-STA results with some representative samples before running a large number of samples.
3. **Assay selection:** Because a universal thermal condition is used in HT-qPCR, assays should have similar Tm values (i.e., within 2°C of the annealing temperature) to avoid nonspecific amplification. We generally recommend using TaqMan probe-assays because they offer higher sensitivity and specificity than dye-based assays (46, 47). Dye-based assays (e.g., ARG assays) can be used for HT-qPCR, but assays should be carefully designed to minimize the detection of non-target amplification.
4. **Primer and probe concentrations:** The primer and probe concentrations for assays should be individually optimized on each HT-qPCR platform. In most cases, the same primer and probe concentrations used for conventional qPCR should work for HT-qPCR, but for some assays, higher concentrations of primer or probes are necessary to be detected on a HT-qPCR platform. In addition, the use of double-quenching probes (e.g., ZEN/Iowa Black) or minor groove binder (MGB) are recommended for HT-qPCR.
5. **Platform considerations:** Both Biomark HD and SmartChip platforms can provide comparable results. The Biomark HD platform uses predefined microfluidic chip formats with sample × assay combinations of 12 × 12, 48 × 48, 96 × 96, 24 × 192, and 192 × 24, whereas the SmartChip system allows greater flexibility in customizing the number of samples and assays analyzed (i.e., 14 different sample × assay combinations, with up to 384 assays or samples on each chip). Because the SmartChip system relies on a multi-sample nanoliter dispenser to load samples and assays onto the chip, whereas the Biomark HD system uses chips with integrated microfluidic channels, the SmartChip MyDesign Chip is less expensive than the Biomark Dynamic Array IFCs, resulting in a lower overall per-run cost (Table S3). However, because the Biomark Dynamic Array supports a greater number of reactions per run (9,216) than the SmartChip MyDesign Chip (5,184), the estimated cost per reaction is relatively similar between the two platforms. Overall, while the prorated cost per reaction of approximately $0.20 is lower than the estimated cost of conventional qPCR ($0.50–$1.50 per reaction; [34]), the per-run cost of approximately $900–$1,900 is considerably higher than that of conventional qPCR. Therefore, users may need to combine multiple samples and/or assays in a single run to fully utilize all wells on the chip and achieve cost-effective operation.
  When using comparable configurations (i.e., a 96.96 Dynamic Array IFC for the Biomark HD and a 54-assay × 96-sample format for the SmartChip), the total time required for experiment preparation and thermal cycling was approximately 5 h for both platforms. However, the SmartChip dispensing system may require additional hands-on time for system setup, as well as more frequent routine maintenance and service visits, than the Biomark HD system. Therefore, the choice of the HT-qPCR platform depends on the number of samples and assays, how frequently you run the chips, and your budget.

In this study, dPCR was used primarily for assay troubleshooting, visualization of endpoint fluorescence patterns, and evaluation of whether specific assays or samples exhibited suboptimal amplification efficiency. Quantitative results generated by dPCR were not directly compared with those from conventional qPCR or HT-qPCR, as such comparisons were beyond the scope of this study. Nevertheless, numerous studies have compared dPCR and qPCR and demonstrated that dPCR offers several advantages, including absolute quantification, high sensitivity for low-abundance targets, and increased tolerance to PCR inhibitors (56–58).

While dPCR is becoming increasingly popular for wastewater surveillance (56, 59, 60) and infectious disease diagnostics, oncology, and genetic testing (58, 61, 62), its multiplex detection capability is limited (e.g., usually up to five targets per reaction; [63]). Therefore, the advantages of HT-qPCR cannot be replaced by dPCR. Instead, HT-qPCR and dPCR can be used for different application purposes. HT-qPCR is suitable for the quantitative screening of target genes across many samples, while dPCR should be used when accurate and absolute quantification is needed for a limited number of targets. The application of multiplex dPCR (i.e., ≥5 targets per reaction) in MST and environmental monitoring remains an important area for future research. Highly multiplexed reactions may introduce challenges such as competition for reaction components (e.g., dNTPs and polymerase), reduced amplification efficiency, and fluorescence signal damping, all of which could affect assay performance and quantification accuracy.

### Conclusions

HT-qPCR is a valuable tool that enables the simultaneous quantification of numerous targets across large numbers of samples. In this study, we demonstrated that quantitative HT-qPCR results generated by two different HT-qPCR platforms (i.e., SmartChip and BioMark HD) were directly comparable. These findings support that quantitative HT-qPCR results generated using different platforms and across different environmental surveillance programs can be compared and integrated in meta-analyses.

However, we also observed differences in the performance of some assays and in binary detection frequencies between the two platforms when using a universal detection threshold (i.e., 100 copies/µL). These discrepancies highlight the importance of careful assay selection, optimization, and validation when comparing results across HT-qPCR platforms. We hope that the general framework reported in this study is helpful for current and future users to design and select appropriate assays for HT-qPCR.

## Data Availability

Data used in this study are available upon request.

## SUPPLEMENTAL MATERIAL

Supplemental material aem.01222-26-s0001.pdf Tables S1 to S3; Fig. S1 to S7.

ASM does not own the copyrights to Supplemental Material that may be linked to, or accessed through, an article. The authors have granted ASM a non-exclusive, world-wide license to publish the Supplemental Material files. Please contact the corresponding author directly for reuse.
