# Supplementary material for: Comparative evaluation of two high-throughput qPCR systems for environmental and clinical microbiology applications
Source: Appl Environ Microbiol. 2026 Sep 25;92(10):e01222-26. doi: 10.1128/aem.01222-26 (PMC13689234; doi:10.1128/aem.01222-26)
Supplement: Supplementary file 1 [file aem.01222-26-s0001.pdf]

Supplementary Materials

Comparative Evaluation of the Two High-Throughput qPCR Systems for Environmental and Clinical Microbiology Applications

Hao Wang[1] and Satoshi Ishii[1, 2*]

[1] BioTechnology Institute, University of Minnesota, St. Paul, MN, USA; 55108
[2] Department of Soil, Water, and Climate, University of Minnesota, St. Paul, MN, USA; 55108

*Corresponding author: ishi0040@umn.edu

This file contains three tables, seven figures, and reference.

Table S1. Primer and probe sequences used in this study to quantify microbial source tracking markers, Fecal Indicator Bacteria, and Pathogens.

Target Organism	Assay Name	Primer/Probe Name	Sequence (5' --> 3')	Reference
General Bacteroides				
Bacteroides spp.	AllBac	AllBac296f	GAGAGGAAGGTCCCCCAC	1
		AllBac412r	CGCTACTTGGCTGGTTCAG	
		AllBac375Bhqr	**FAM-**CCATTGACC**/ZEN/**AATATTCCTCACTGCTGCCT**-IBFQ**	
	BacGeneral	BacGen-F	CTGAGAGGAAGGTCCCCCAC	2
		BacGen-R	CACGCTACTTGGCTGGTTCAG	
		BacGen-TP	**FAM-**AGCAGTGAGGAATATT**-MGB-NFQ**	
Human				
Human *Bacteroides*	HF183	HF183	ATCATGAGTTCACATGTCCG	3
		BacR287	CTTCCTCTCAGAACCCCTATCC	
		BacP234MGB	**FAM-**CTAATGGAACGCATCCC**-MGB-NFQ**	
	gyrB	Bf904F	GGCGGTCTTCCGGGTAAA	4
		Bf1272R	TGGCATATAGCGGAAGAAAAAAG	
		Bf923MGB	**FAM-**TGGCCGACTGCTC**-MGB-NFQ**	
	HumM2	Hum2F	CGTCAGGTTTGTTTCGGTATTG	5
		Hum2R	TCATCACGTAACTTATTTATATGCATTAGC	
		Hum2P	**FAM-**TATCGAAAA**/ZEN/**TCTCACGGATTAACTCTTGTGTACGC**-IBFQ**	
Human-specific *E. coli*	H8	H8_Forward	ACAGTCAGCGAGATTCTTC	6, 7
		H8_Reverse	GAACGTCAGCACCACCAA	
		H8_Probe	**FAM-**ACTGGCATC**/ZEN/**GGCATGGAACAC**-IBFQ**	
	H12	H12_Forward	GTAAAAGGACTGCCGGGAAA	6, 7
		H12_Reverse	TCAGATCGTCCTTTACCAG	
		H12_Probe	**FAM-**AGAGTAAAC**/ZEN/**GCTTCGCCCTTAGCC**-IBFQ**	

Table S1. Continued.

Target Organism	Assay Name	Primer/Probe Name	Sequence (5' --> 3')	Reference
Methanobrevibacter smithii nifH	Mnif	Mnif_202F	GAAAGCGGAGGTCCTGAA	8
		Mnif_353R	ACTGAAAAACCTCCGCAAAC	
		Mnif Probe	**FAM-**CCGGACGTG/**ZEN**/GTGTAACAGTAGCTA**-IBFQ**	
crAssphage	CPQ_056	056F1	CAGAAGTACAAACTCCTAAAAAACGTAGAG	9
		056R1	GATGACCAATAAACAAGCCATTAGC	
		056P1	**FAM-**AATAACGATTTACGTGATGTAAC**-MGB-NFQ**	
	CPQ_064	064F1	TGTATAGATGCTGCTGCAACTGTACTC	9
		064R1	CGTTGTTTTCATCTTTATCTTGTCCAT	
		064P1	**FAM-**CTGAAATTGTTCATAAGCAA**-MGB-NFQ**	
Human mtDNA	Human-ND5	Human-ND5_forward	CAGCAGCCATTCAAGCAATGC	10
		Human-ND5_reverse	GGTGGAGACCTAATTGGGCTGATTAG	
		Human-ND5_probe	**FAM-**TATCGGCGA/**ZEN**/TATCGGTTTCATCCTCG**-IBFQ**	
Dog				
Dog *Bacteroides*	BacCan-UCD	BacCan-545f1	GGAGCGCAGACGGGTTTT	11
		BacUni-690r1	CAATCGGAGTTCTTCGTGATATCTA	
		BacUni-690r2	AATCGGAGTTCCTCGTGATATCTA	
		BacUni-656p	**FAM-**TGGTGTAGCGGTGAAA**-MGB-NFQ**	
	DogBact	DF475F	CGCTTGTATGTACCGGTACG	12, 13
		Bac708R	CAATCGGAGTTCTTCGTGAT	
		DogBactP	**FAM-**ATTCGTGGT/**ZEN**/GTAGCGGTGAAATGCTTAG**-IBFQ**	
Dog mtDNA	Dog-ND5	Dog-ND5_forward	GGCATGCCTTTCCTTACAGGATTC	14
		Dog-ND5_reverse	GGGATGTGGCAACGAGTGTAATTATG	
		Dog-ND5_probe	**FAM-**TCATCGAGT/**ZEN**/CCGCTAACACGTCGAAT**-IBFQ**	

Table S1. Continued.

Target Organism	Assay Name	Primer/Probe Name	Sequence (5' --> 3')	Reference
Cow				
Cow *Bacteroides*	CowM2	CowM2F	CGGCCAAATACTCCTGATCGT	15
		CowM2R	GCTTGTTGCGTTCCTTGAGATAAT	
		CowM2P	**FAM-**AGGCACCTA/**ZEN**/TGTCCTTTACCTCATCAACTACAGACA-**IBFQ**	
	CowM3	CowM3F	CCTCTAATGGAAAATGGATGGTATCT	15
		CowM3R	CCATACTTCGCCTGCTAATACCTT	
		CowM3P	**FAM-**TTATGCATT/**ZEN**/GAGCATCGAGGCC-**IBFQ**	
	BacBov1	BacBov-F1	AAGGATGAAGGTTCTATGGATTGTAAA	2
		BacBov-R1	GAGTTAGCCGATGCTTATTCATACG	
		BacBov-TP1	**FAM**-ATACGGGAATAAAACC-**MGB-NFQ**	
	BacBov2	BacBov-F2	GGATTACAGCCCTACGGGTTTTA	2
		BacBov-R2	GGAGTTAGCCGATGCTTATTCATATAA	
		BacBov-TP2	**FAM**-AAGAGTAATGTGCACTACGTG-**MGB-NFQ**	
Cow mtDNA	Cow-ND5	Cow-ND5_forward	CAGCAGCCCTACAAGCAATGT	10
		Cow-ND5_reverse	GAGGCCAAATTGGGCGGATTAT	
		Cow-ND5_probe	**FAM-**CATCGGCGA/**ZEN**/CATTGGTTTCATTTTAG-**IBFQ**	

Table S1. Continued.

Target Organism	Assay Name	Primer/Probe Name	Sequence (5' --> 3')	Reference
Ruminant				
Ruminant *Bacteroides*	BacR	BacR_f	GCGTATCCAACCTTCCCG	16
		BacR_r	CATCCCCATCCGTTACCG	
		BacR_p	**FAM-**CTTCCGAAAGGGAGATT**-MGB-NFQ**	
	Run-2-Bac	BacB2-590F	ACAGCCCGCGATTGATACTGGTAA	17
		Bac708Rm	CAATCGGAGTTCTTCGTGAT	
		BacB2-626P	**FAM**-ATGAGGTGG/**ZEN**/ATGGAATTCGTGGTGT-**IBFQ**	
Pig				
Pig *Bacteroides*	Pig-1-Bac	Pig-1-Bac32Fm	AACGCTAGCTACAGGCTTAAC	18
		Pig-1-Bac108R	CGGGCTATTCCTGACTATGGG	
		Pig-1-Bac44P	**FAM**-ATCGAAGCT/**ZEN**/TGCTTTGATAGATGGCG-**IBFQ**	
	Pig-2-Bac	Pig-2-Bac41F	GCATGAATTTAGCTTGCTAAATTTGAT	18
		Pig-2-Bac163R	ACCTCATACGGTATTAATCCGC	
		Pig-2-Bac113P	**FAM**-TCCACGGGATAGCC-**MGB-NFQ**	
Pig mtDNA	Pig-ND5	Pig-ND5_forward	ACAGCTGCACTACAAGCAATGC	10
		Pig-ND5_reverse	GGATGTAGTCCGAATTGAGCTGATTAT	
		Pig-ND5_probe	**FAM-**CATCGGAGA**/ZEN/**CATTGGATTTGTCCTAT**-IBFQ**	
Avian				
Lactobacillus spp.	Av4143	Av4143F	TGCAAGTCGAACGAGGATTTCT	19
		Av4143R	TCACCTTGGTAGGCCGTTACC	
		Av4143P	**FAM-**AGGTGGTTT/**ZEN**/TGCTATCGCTTT**-IBFQ**	

Table S1. Continued.

Target Organism	Assay Name	Primer/Probe Name	Sequence (5' --> 3')	Reference
Fecal indicator bacteria				
Enterococcus spp.	Entero1	ECST748F	GAGAAATTCCAAACGAACTTG	20
		ENC854R	CAGTGCTCTACCTCCATCATT	
		GPL813TQ	**FAM-**TGGTTCTCT**/ZEN/**CCGAAATAGCTTTAGGGCTA**-IBFQ**	
E. coli	*uidA*	uidA405F	CAACGAACTGAACTGGCAGA	21
		uidA525R	CATTACGCTGCGATGGAT	
		uidA429P	**FAM-**CCCGCCGGG**/ZEN/**AATGGTGATTAC**-IBFQ**	
Bacterial pathogens				
Pathogenic *E. coli*	*stx*$_1$	VS1 (Forward)	CATAGTGGAACCTCACGACGCAGT	22
		VS2 (Reverse)	TTTGCCGAAAACGTAAAGCTTCA	
		VS3 (Probe)	**FAM-**TGTGGCAAG**/ZEN/**AGCGATGTTACGGTTTG**-IBFQ**	
	stx$_2$	Stx2F	ACCACATCGGTGTCTGTTATTAACC	23
		Stx2R	CGGTAGAAAGTATTTGTTGCCGTATT	
		Stx2P	**FAM-**TTTGCTGTG**/ZEN/**GATATACGAGGGCTTGATGTCTAT**-IBFQ**	
	eaeA	VS8 (Forward)	GGCGGATTAGACTTCGGCTA	22
		VS9 (Reverse)	CGTTTTGGCACTATTTGCCC	
		VS10 (Probe)	**FAM-**AACGCCGAT**/ZEN/**ACCATTACTTATACCGCGACG**-IBFQ**	
Shigella spp.	*ipaH*	ipaH-U1	CCTTTTCCGCGTTCCTTGA	24
		ipaH-L1	CGGAATCCGGAGGTATTGC	
		ipaH_Probe	**FAM-**CGCCTTTCC**/ZEN/**GATACCGTCTCTGCA**-IBFQ**	
	virA	Forward	CTGCATTCTGGCAATCTCTT	25
		Reverse	GGGACAACTGCGTTGATTTT	
		virA_Probe	**FAM-**TGCCCAAAT**/ZEN/**TATGTCCGAAAACA**-IBFQ**	

Table S1. Continued.

Target Organism	Assay Name	Primer/Probe Name	Sequence (5' --> 3')	Reference
Salmonella spp.	*invA*	invA_176F	CAACGTTTCCTGCGGTACTGT	26
		invA_291R	CCCGAACGTGGCGATAATT	
		invA_Tx_208	**FAM-**CTCTTTCGT/**ZEN**/CTGGCATTATCGATCAGTACCA-**IBFQ**	
	ttr	ttr-6_forward	CTCACCAGGAGATTACAACATGG	27
		ttr-4_reverse	AGCTCAGACCAAAAGTGACCATC	
		ttr-5	**FAM-**CACCGACGG**/ZEN/**CGAGACCGACTTT**-IBFQ**	
Campylobacter jejuni	*ciaB*	ciaB_Forward	CAACTTTATATTTGCACTCCGATG	28
		ciaB_Reverse	GGAACGACTTGAGCTGAGAATAAAC	
		ciaB_Probe	**FAM-**TATGGTGCTGAACTTAAA**-MGB-NFQ**	
	hipO	CJ_hipO_F	AATGCACAAATTTGCCTTATAAAAGC	29
		CJ_hipO_R	TNCCATTAAAATTCTGACTTGCTAAATA	
		CJ_hipO_probe	**FAM-**ACATACTACTTCTTTATTGCTTG**-MGB-NFQ**	
Campylobacter coli	*cadF*	CC_cadF_F	GAGAAATTTTATTTTTATGGTTTAGCTGGT	29
		CC_cadF_R	ACCTGCTCCATAATGGCCAA	
		CC_cadF_probe	**FAM-**CCTCCACTT**/ZEN/**TTATTATCAAAAGCGCCTTTAGAAA**-IBFQ**	
Clostridium perfringens	*cpe*	cpe F	AACTATAGGAGAACAAAATACAATAG	30
		cpe R	TGCATAAACCTTATAATATACATATTC	
		cpe Pr	**FAM-**TCTGTATCT**/ZEN/**ACAACTGCTGGTCCA**-IBFQ**	
	CPerf16S	CPerf165F	CGCATAACGTTGAAAGATGG	31
		CPerf269R	CCTTGGTAGGCCGTTACC	
		CPerf187FAM	**FAM**-TCATCATTC/ZEN/AACCAAAGGAGCAATCC-**IBFQ**	
Listeria monocytogenes	*iap*	iap (forward)	CTCAAATACGAATGCTAACCAAGGT	32
		iap (reverse)	TTTGAGCTTCAGCAATAATAGCACTT	
		iap (probe)	**FAM-**TAACAGCAATTCAAGCGC**-MGB-NFQ**	

Table S1. Continued.

Target Organism	Assay Name	Primer/Probe Name	Sequence (5' --> 3')	Reference
Listeria monocytogenes	*hlyA*	hlyA-LisM-F	ACTGAAGCAAAGGATGCATCTG	33
		hlyA-LisM-R	TTTTCGATTGGCGTCTTAGGA	
		hlyA-LisM-P	**FAM-**CACCACCAG**/ZEN/**CATCTCCGCCTGC**-IBFQ**	
Vibrio cholerae	*ctxA*	VC_ctxAF	TTTGTTAGGCACGATGATGGAT	34
		VC_ctxAR	ACCAGACAATATAGTTTGACCCACTAAG	
		VC_ctxA_probe	**FAM-**TGTTTCCAC**/ZEN/**CTCAATTAGTTTGAGAAGTGCCC**-IBFQ**	
	toxR	VC_toxR_420/334F	GTTTGGCGWGAGCAAGGTTT	35, 36
		VC_toxR_585R	TCTCTTCTTCAACCGTTTCCA	
		toxR_464/378FAM	**FAM**-CGCAGAGTMGAAATGGCTTGG-**MGB-NFQ**	
Vibrio parahaemolyticus	*tdhS*	VP_tdhF	AAACATCTGCTTTTGAGCTTCCA	37
		VP_tdhR	CTCGAACAACAAACAATATCTCATCAG	
		VP_tdhS	**FAM**-TGTCCCTTT/**ZEN**/TCCTGCCCCCGG-**IBFQ**	
Legionella spp.	*ssrA*	PanLeg-F	GGCGACCTGGCTTC	38
		PanLeg-R1	GGTCATCGTTTGCATTTATATTTA	
		PanLeg-P1	**FAM-**CGTGGGTTGCAA**-MGB-NFQ**	
L. pneumophila	*mip*	Lp-F	TTGTCTTATAGCATTGGTGCCG	38
		Lp-R	CCAATTGAGCGCCACTCATAG	
		Lp-P	**FAM-**CGGAAGCAA**/ZEN/**TGGCTAAAGGCATGCA**-IBFQ**	
L. pneumophila serogroup 1	*wzm*	Lp1-F	TGCCTCTGGCTTTGCAGTTA	38
		Lp1-R	CACACAGGCACAGCAGAAACA	
		Lp1-P	**FAM-**TTTATTACTCCACTCCAGCGAT**-MGB-NFQ**	
Mycobacterium spp.	*atpE*	FatpE	CGGYGCCGGTATCGGYGA	39
		RatpE	CGAAGACGAACARSGCCAT	
		PatpE	**FAM-**ACSGTGATG**/ZEN/**AAGAACGGBGTRAA**-IBFQ**	

Table S1. Continued.

Target Organism	Assay Name	Primer/Probe Name	Sequence (5' --> 3')	Reference
M. avium complex	MAC ITS	MAC_Forward	TTGGGCCCTGAGACAACACT	40
		MAC_Reverse	GCAACCACTATCCAATACTCAAACAC	
		MAC_Probe	**FAM-**CCGTGTGGA/**ZEN**/GTCCCTCCATCTTGG**-IBFQ**	
Protozoan pathogens				
Giardia sp.	beta-Giardin P241	P241_Forward	CATCCGCGAGGAGGTCAA	41
		P241_Reverse	GCAGCCATGGTGTCGATCT	
		P241_Probe	**FAM-**AAGTCCGCC/**ZEN**/GACAACATGTACCTAACGA**-IBFQ**	
Cryptosporidium spp.	COWP P702	P702_Forward	CAAATTGATACCGTTTGTCCTTCTG	41
		P702_Reverse	GGCATGTCGATTCTAATTCAGCT	
		P702_Probe	**FAM-**TGCCATACA/**ZEN**/TTGTTGTCCTGACAAATTGAAT**-IBFQ**	
Entamoeba histolytica	Ehf	Ehf	AACAGTAATAGTTTCTTTGGTTAGTAAAA	42
		Ehr	CTTAGAATGTCATTTCTCAATTCAT	
		Ehp	**FAM-**ATTAGTACA/**ZEN**/AAATGGCCAATTCATTCA**-IBFQ**	
Acanthamoeba spp.	Acant	AcantF900	CCCAGATCGTTTACCGTGAA	43
		AcantR1100	TAAATATTAATGCCCCCAACTATCC	
		AcantP1000	**FAM-**CTGCCACCG/**ZEN**/AATACATTAGCATGG**-IBFQ**	
Naegleria fowleri	Naegl	NaeglF192	GTGCTGAAACCTAGCTATTGTAACTCAGT	43
		NaeglR344	CACTAGAAAAAGCAAACCTGAAAGG	
		NfowlP	**FAM-**ATAGCAATA/**ZEN**/TATTCAGGGGAGCTGGGC**-IBFQ**	

Table S2. Primer sequences used in this study to quantify antibiotic and heavy metal resistance genes.

Target Gene	Sequence (5' --> 3')	Target Function	Reference
aacA	GTGTAACACGCAAGCACGAT<br>AGCCTCCGCGATTTCATAC	aminoglycoside resistance	44
aadA5	ATCTTGCGATTTTGCTGACC<br>TGTACCAAATGCGAGCAAGA	animoglycoside resistance	44
aadD	ATGGGGATGATGTTAAGGCT<br>TCACTTCCACCTTCCACTCA	kanamycin resistance	44
acrD	GGCAATCCTGTTGTGTCTGA<br>ACATGAGATTATCGAGGCCG	multidrug efflux pumps	44
ampC	CCTCTTGCTCCACATTTGCT<br>ACAACGTTTGCTGTGTGACG	penicillin/cephalospiron resistance	44
arr2	TTACAAGCAGGTGCAAGGAC<br>GCTCCATCAAGGCTGAAAAG	Rifampicin resistance	44
$bla_{ctx\text{-}m\text{-}32}$	CGTCACGCTGTTGTTAGGAA<br>CGCTCATCAGCACGATAAAG	class A beta-lactamase resistance	44
$bla_{imp\text{-}13}$	AGGAGCGGCTTTACCTGATT<br>CGCTCCACAAACCAATTGAC	class B beta-lactamase resistance	44
bla_{KPC}	GATACCACGTTCCGTCTGG<br>GCAGGTTCCGGTTTTGTCTC	beta-lactamase resistance	45
$bla_{NDM\text{-}1}$	ATTAGCCGCTGCATTGAT<br>CATGTCGAGATAGGAAGTG	beta-lactamase resistance	46

Table S2. Continued.

Target Gene	Sequence (5' --> 3')	Target Function	Reference
*bla*NPS	GGACCATCGTCATCGAGTCT<br>ATTCGCAATCGAATACTGGG	class D beta-lactamase resistance	44
*bla*OXA	TGATGATTGTCGAAGCCAAA<br>GCCTGTAGGCCACTCTACCC	class D beta-lactamase resistance	47
*bla*SHV	AACGGAACTGAATGAGGCGCT<br>TCCACCATCCACTGCAGCAGCT	class A beta-lactamase resistance	48
*bla*VIM	CGCAGCTTTCTGGTTGGTAT<br>CGTGTCACCGAGTTTCTGAG	class B beta-lactamase resistance	44
cadA	GTGAGCAGGCCAGCACTGAA<br>TCTCGATGCGGTAGGTGGTC	cadmium resistance	49
catB8	GGGGAACTTCTTTCTGAGCA<br>CCCCGCTTCCTATAGAACAA	chloramphenicol resistance	44
chrA	TCACGCCGGAATATAACTAC<br>CGTACCCTGATCAATCACTT	chromium resistance	50
cmlB	TAATTGGCGGTATTCCCTTG<br>AGCGTAAGCTGAATTGTGCC	Chloramphenicol/florfenicol transporters	44
copA	GTGTACGGTCCGCTGGTTAT<br>CTTGAACACTCCGGTCCAG	copper resistance	51
dfr13	AATCGGTCCGCATTTATCTG<br>TTGGTAAGGGCTTGCCTATG	trimethoprim resistance	44

Table S2. Continued.

Target Gene	Sequence (5' --> 3')	Target Function	Reference
ereB	TCTGCATTATGCCAACGGTA<br>TCTGCTCACTTTGTGGGTTTT	esterase resistance	44
ermB	GATACCGTTTACGAAATTGG<br>GAATCGAGACTTGAGTGTGC	erythromycin resistance	52
ermF	CGACACAGCTTTGGTTGAAC<br>GGACCTACCTCATAGACAAG	erythromycin resistance	53
floR	TCGTCATCTACGGCCTTTTC<br>CTTGACTTGATCCAGAGGGC	chloramphenicol/florfenicol transporters	44
intI1	CCTCCCGCACGATGATC<br>TCCACGCATCGTCAGGC	class 1 integrons	54
intI2	GACGGCTACCCTCTGTTATCTC<br>TGCTTTTCCCACCCTTACC	class 2 integrons	55
intI3	GGATGTCTGTGCCTGCTTG<br>GCCACCACTTGTTTGAGGA	class 3 integrons	55
mefE	CCTGCAAATGGCGATTATTT<br>AATAGCAAGCACTGCACCAG	macrolide efflux proteins	44
merA	GTGCCGTCCAAGATCATG<br>GGTGGAAGTCCAGTAGGGTGA	mercury resistance	56
mexB	GTGTTCGGCTCGCAGTACTC<br>AACCGTCGGGATTGACCTTG	multidrug efflux pump	57

Table S2. Continued.

Target Gene	Sequence (5' --> 3')	Target Function	Reference
nikA	AATATCAGGCAGACGGTTCG<br>AGGGTGAAGGTCCAGGTTTT	nickel resistance	58
qacF	TGGCTGTTTCAATCTTTGGC<br>GCCCATACAGCGTAAGCAAT	quaternary ammonium compounds resistance	44
qnrA	AGGATTTCTCACGCCAGGATT<br>CCGCTTTCAATGAAACTGCA	quinolone resistance	59
qnrB	AAATATGGCTCTGGCACTCG<br>CTTTCAGCATCGCACGACTA	quinolone resistance	44
rcnA	GGGCACTCAAAAACGATGAT<br>GCGAAATAGTTGCTGCCAGT	nickel and cobalt resistance	58
strB	GCCTGTTTTTCCTGCTCATT<br>CGCAGTTCATCAGCAATGTC	streptomycin resistance	44
sul1	CCGTTGGCCTTCCTGTAAAG<br>TTGCCGATCGCGTGAAGT	sulfonamide resistance	60
sul2	GACAGTTATCAACCCGCGAC<br>GTCTTGCACCGAATGCATAA	sulfonamide resistance	44
sul3	TCCGTTCAGCGAATTGGTGCAG<br>TTCGTTCACGCCTTACACCAGC	sulfonamide resistance	61
tet(A)	GCTACATCCTGCTTGCCTTC<br>CATAGATCGCCGTGAAGAGG	tetracycline resistance	62

Table S2. Continued.

Target Gene	Sequence (5' --> 3')	Target Function	Reference
tet(L)	TCGTTAGCGTGCTGTCATTC<br>GTATCCCACCAATGTAGCCG	tetracycline resistance	62
tet(M)	GTGGACAAAGGTACAACGAG<br>CGGTAAAGTTCGTCACACAC	tetracycline resistance	62
tet(S)	CAAGGATTGTACGGTTGGAAA<br>TTTCGAAGCTAAGATATGGCTC	tetracycline resistance	44
tet(W)	GAGAGCCTGCTATATGCCAGC<br>GGGCGTATCCACAATGTTAAC	tetracycline resistance	63
tet(X)	AGCCTTACCAATGGGTGTAAA<br>TTCTTACCTTGGACATCCCG	tetracycline resistance	64
vanA	GTAGGCTGCGATATTCAAAGC<br>CGATTCAATTGCGTAGTCCAA	vancomycin resistance	65
vanB	TTGCATGGACAAATCACTGG<br>GCTCGTTTTCCTGATGGATG	vancomycin resistance	66

Table S3. Estimated costs of HT-qPCR runs on the Biomark HD and SmartChip platforms.[a]

Platform	Consumable (Catalog No.)	Cost per run[b]	Cost per reaction[c]
Biomark HD	96.96 DynamicArray IFC (BMK-M-96.96)	$1,546	$0.168
	Control Line Fluid (101-8274)	$20	$0.002
	Sample/Assay loading reagent (100-7609 [EvaGreen], 100-7610 [TaqMan Probe], 100-7611 [EvaGreen/TaqMan Probe])	$70	$0.007
	qPCR mastermix (1725211 [EvaGreen], 1725281 [TaqMan Probe])	$55	$0.006
	Assay (primer/probe/gBlocks)[d]	$192	$0.020
	Total	$1,883	$0.205
SmartChip	SmartChip MyDesign Kit (640032)	$593	$0.110
	qPCR mastermix (640211 [TB Green], 640209 [TaqMan Probe])	$200	$0.039
	Assay (primer/probe/gBlocks)[d]	$108	$0.007
	Total	$901	$0.156

[a] The cost comparison was based on a 96.96 DynamicArray IFC configuration for the Biomark HD platform and a 54-assay × 96-sample configuration for the SmartChip platform. These estimates do not include labor, common laboratory consumables (e.g. 96-well plates, plate seals, and pipette tips), the cost for the pre-amplification reaction, and the costs associated with instrument maintenance and service contracts for the HT-qPCR systems.
[b] Based on the catalog price of the smallest scale of the item for the US customers as of June 14, 2026. If the volume of the reagent is greater than that needed to run a chip, the item price was divided by the number of run supported by the item.
[c] The number of reactions per run used for this calculation is 9,216 and 5,184 for Biomark HD and SmartChip platforms, respectively.
[d] Assay costs are rough estimate. Cost for primers, probes, and synthetic DNA fragments can vary substantially depending on the manufacturer, assay chemistry, target sequence length, purification method, and production scale. The cost for intercalating dye (e.g. EvaGreen and TB Green) assays are slightly lower than that for TaqMan probe assays.

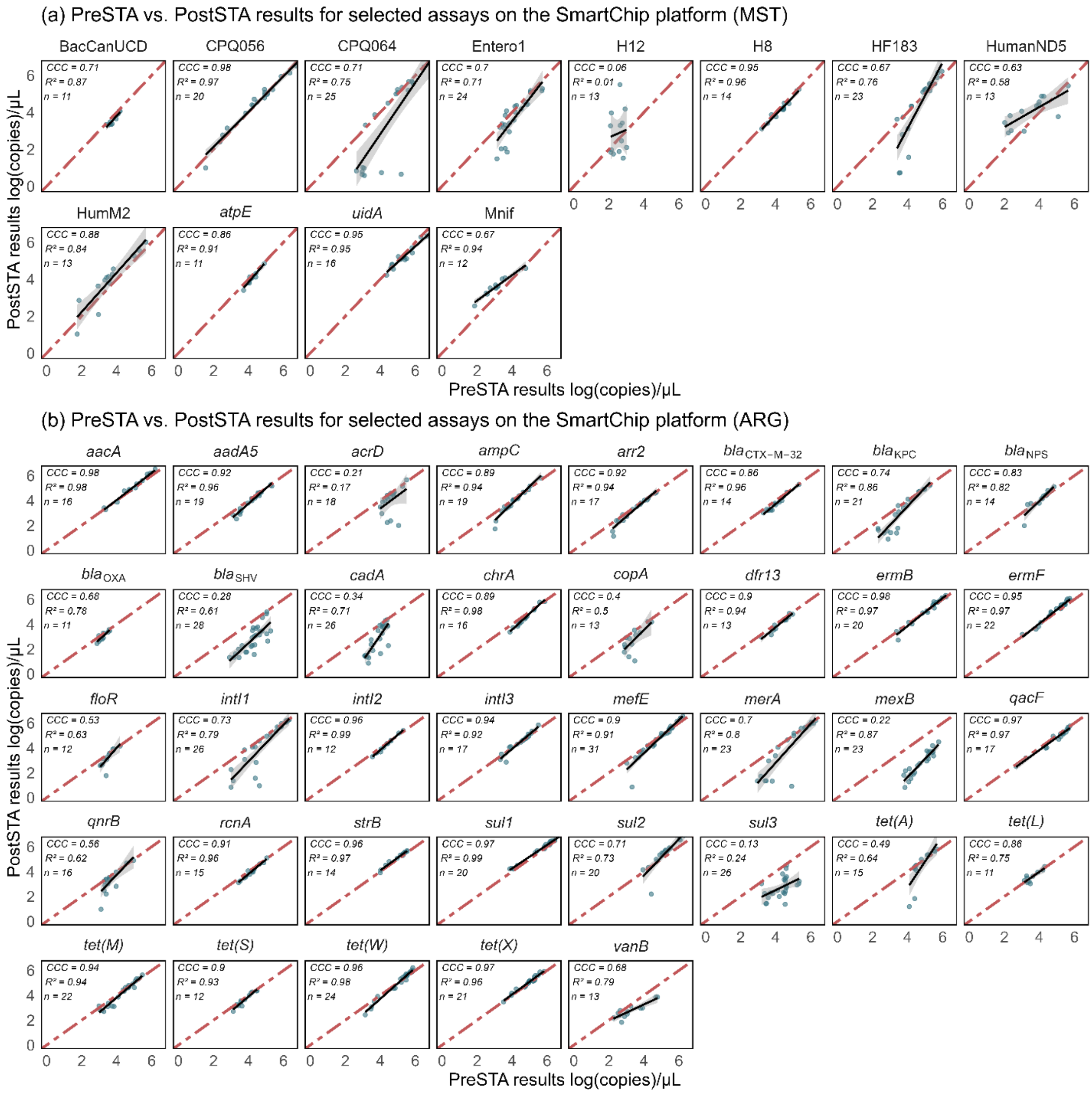

Figure S1. Correlation plots of (a) Microbial source tracking (MST) and (b) Antibiotic Resistance Gene (ARG) assays with more than 10 paired detections in samples before and after specific target amplification (STA) analyzed on the SmartChip platform (Takara Bio). The red dashed line indicates perfect agreement (y = x), the black line represents the linear regression fit, and the gray shaded area represents the 95% confidence interval. Coefficients of determination (R^2) for the fitted linear regression models, Lin's concordance correlation coefficients (CCC), and the number of samples detected are shown for each assay. Most assays exhibited strong quantitative agreement (CCC > 0.7).

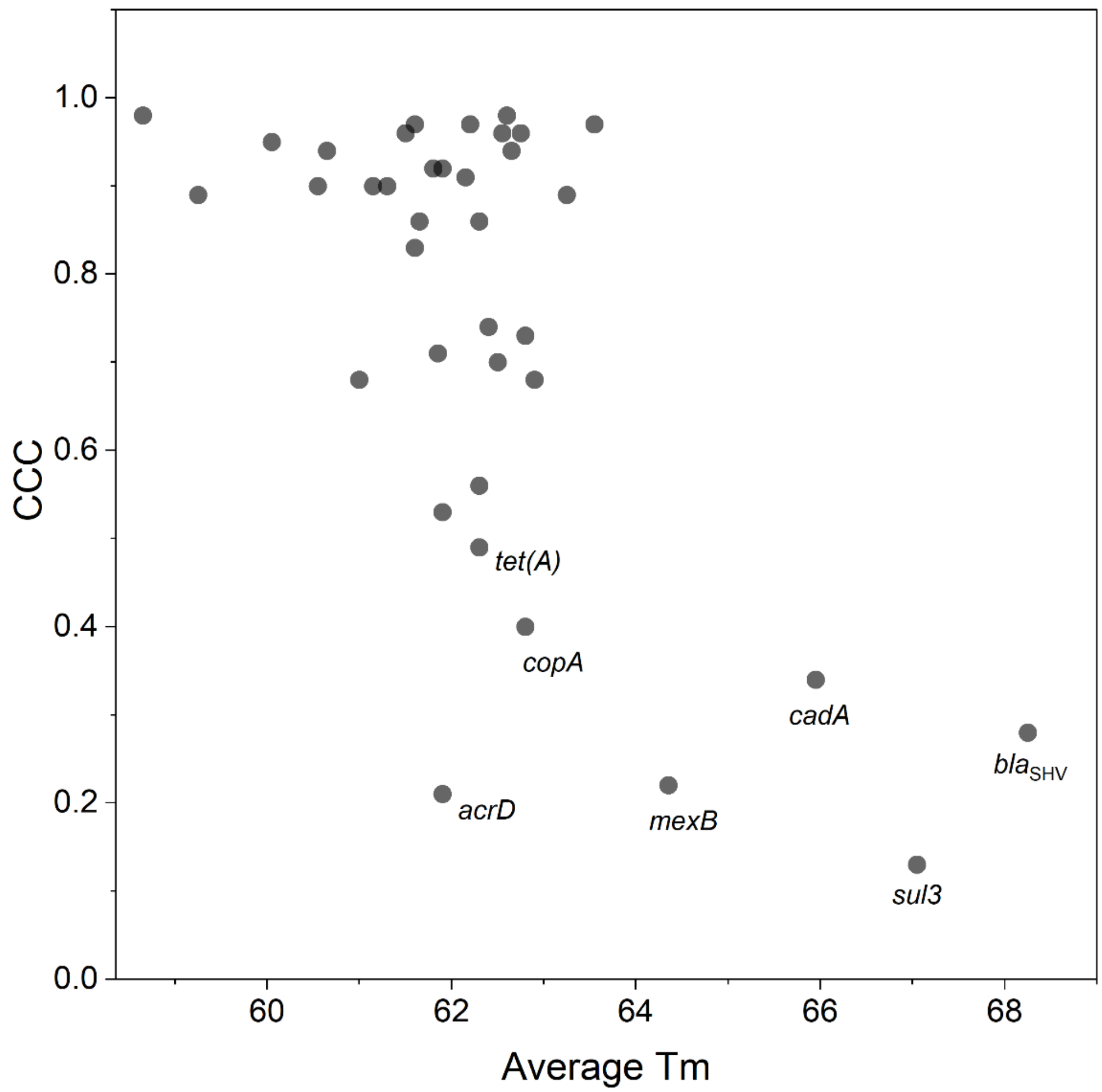

Figure S2. Relationship between the average primer melting temperature (Tm) and Lin's concordance correlation coefficient (CCC) for pre-STA and post-STA measurements of ARG assays with more than 10 paired detections. Assays with CCC values below 0.5 are labeled. Most assays with CCC values below 0.5 had average Tm exceeding 62°C.

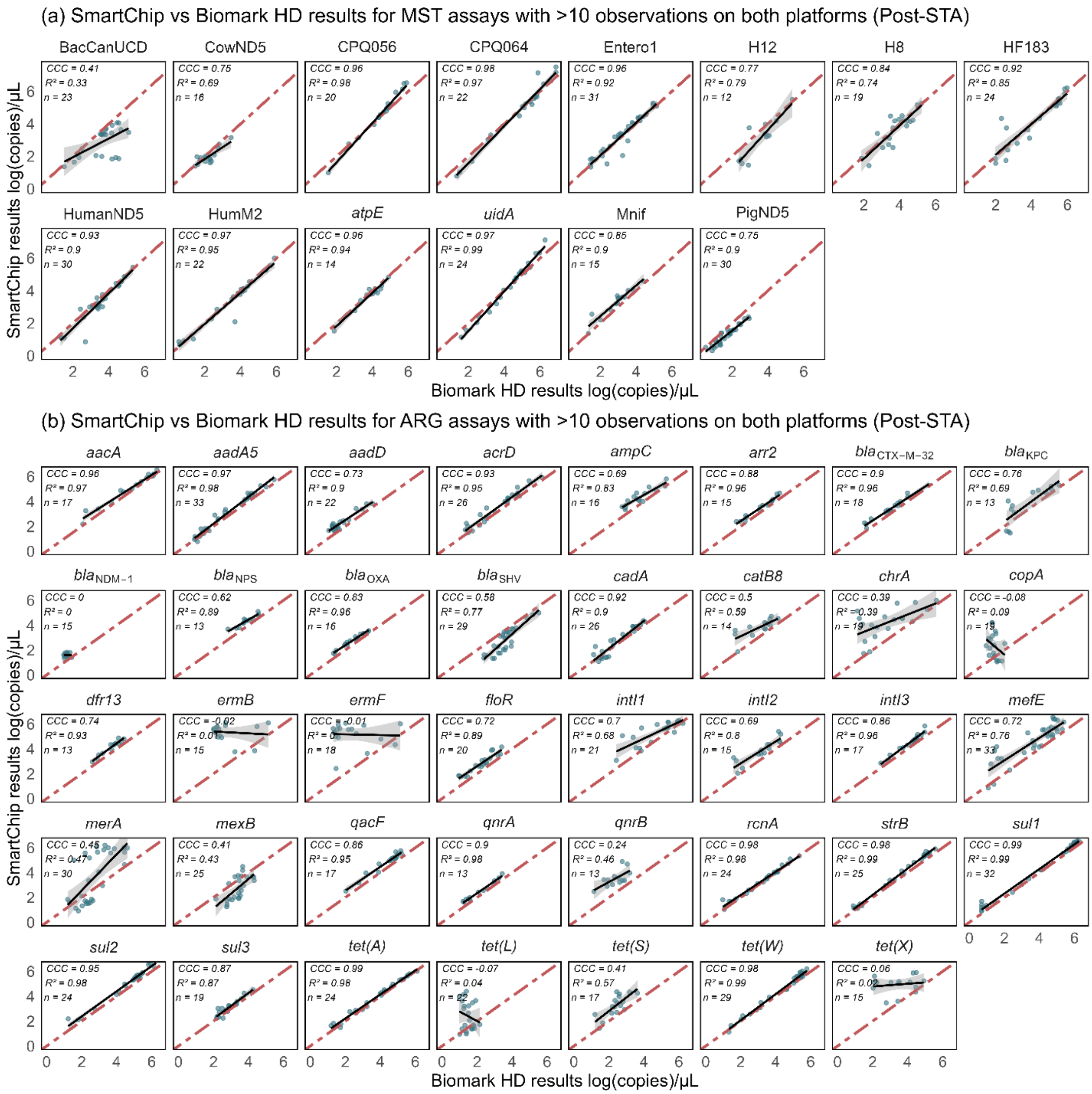

Figure S3. Correlation plots of (a) Microbial source tracking (MST) and (b) Antibiotic Resistance Gene (ARG) assays with more than 10 paired detections on both the SmartChip (Takara Bio) and Biomark HD (Standard BioTools) platforms. The red dashed line indicates perfect agreement (y = x), the black line represents the linear regression fit, and the gray shaded area represents the 95% confidence interval. Coefficients of determination (R^2) for the fitted linear regression models, Lin's concordance correlation coefficients (CCC), and the number of samples detected are shown for each assay. Most assays exhibited strong quantitative agreement (CCC > 0.7).

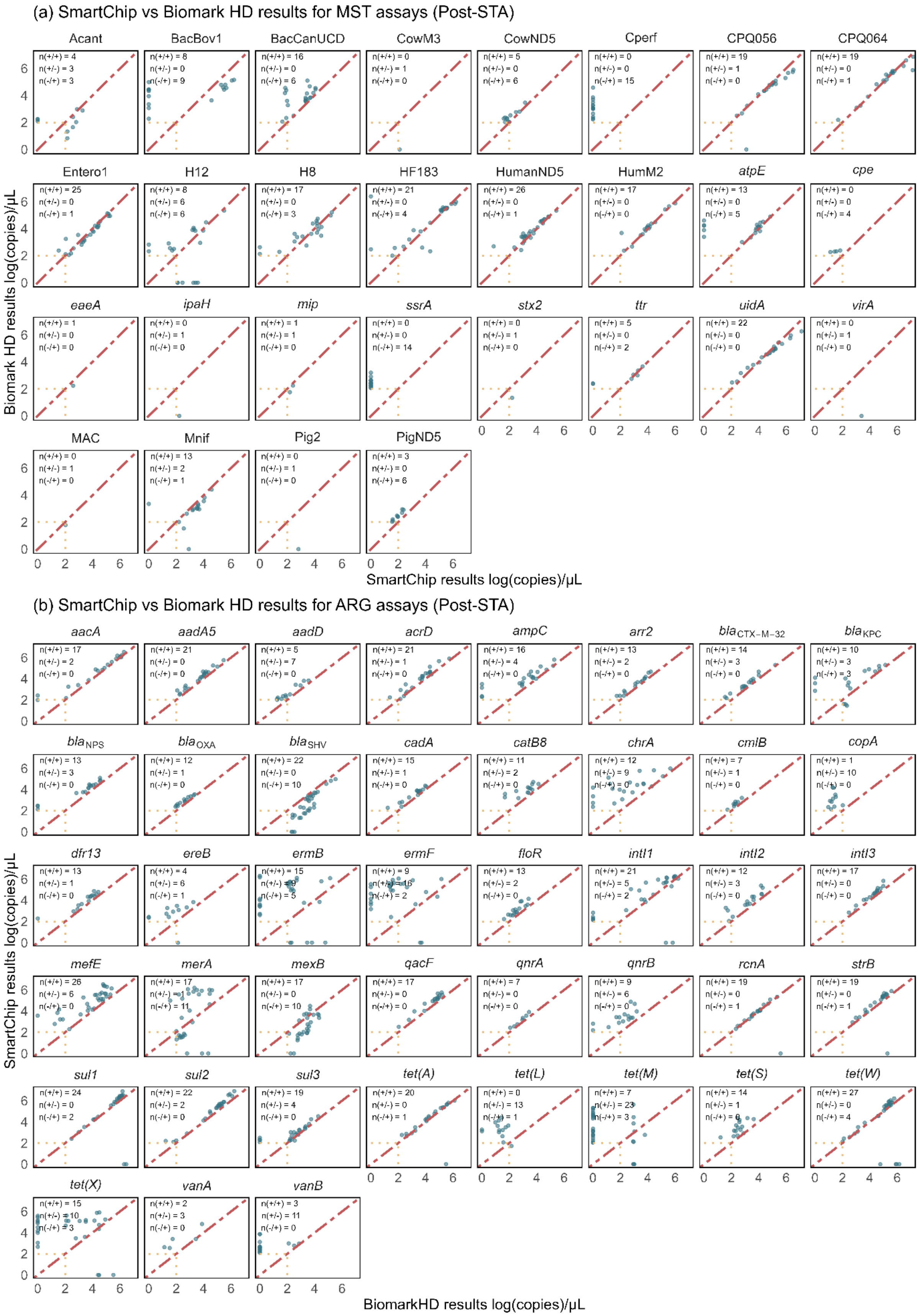
(a) SmartChip vs Biomark HD results for MST assays (Post-STA)
Acant
BacBov1
BacCanUCD
CowM3
CowND5
Cperf
CPQ056
CPQ064
Entero1
H12
H8
HF183
HumanND5
HumM2
atpE
cpe
eaeA
ipaH
mip
ssrA
stx2
ttr
uidA
virA
MAC
Mnif
Pig2
PigND5
Biomark HD results log(copies)/μL
SmartChip results log(copies)/μL
(b) SmartChip vs Biomark HD results for ARG assays (Post-STA)
aacA
aadA5
aadD
acrD
ampC
arr2
blaCTX-M-32
blaKPC
blaNPS
blaOXA
blaSHV
cadA
catB8
chrA
cmlB
copA
dfr13
ereB
ermB
ermF
floR
intI1
intI2
intI3
mefE
merA
mexB
qacF
qnrA
qnrB
rcnA
strB
sul1
sul2
sul3
tet(A)
tet(L)
tet(M)
tet(S)
tet(W)
tet(X)
vanA
vanB
SmartChip results log(copies)/μL
BiomarkHD results log(copies)/μL

Figure S4. Scatter plots of post-STA measurements for samples that tested positive (i.e. >10^2 copies/μL on at least one HT-qPCR platform) for (a) MST assays and (b) ARG assays. Measurements obtained from the Biomark HD and SmartChip platforms are compared. The red dashed line indicates perfect agreement (y = x), and the orange dashed lines indicate the positivity threshold (10^2 copies/μL).

For each assay, n(+/+) represents the number of samples detected above the positivity threshold (>10^2 copies/μL) on both the Biomark HD and SmartChip platforms. n(+/−) represents the number of samples that were positive on the SmartChip platform (>10^2 copies/μL) but negative on the Biomark HD platform (≤10^2 copies/μL). n(−/+) represents the number of samples that were negative on the SmartChip platform (≤10^2 copies/μL) but positive on the Biomark HD platform (>10^2 copies/μL).

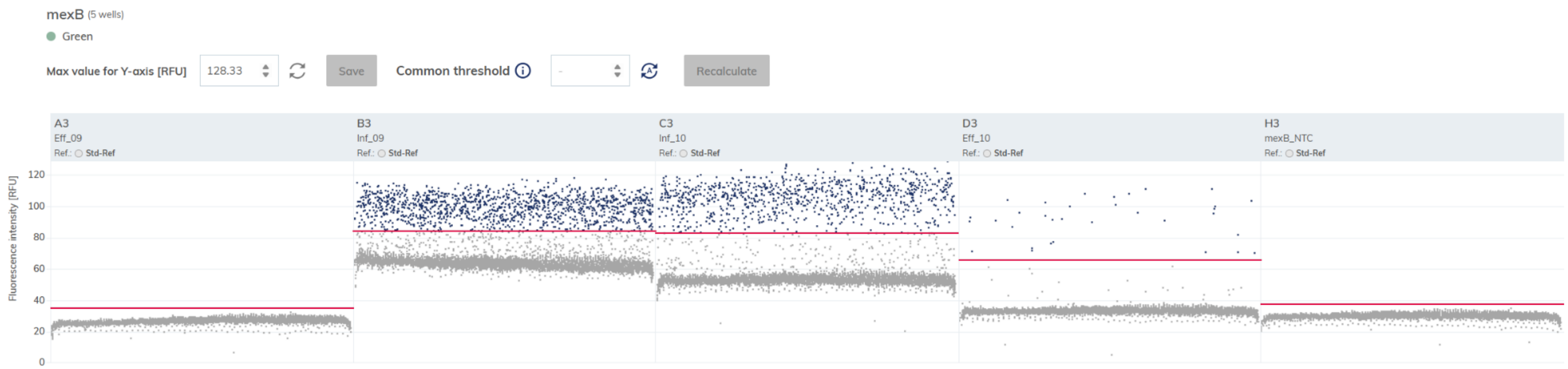

Figure S5. Scatter plots of endpoint fluorescence signals from individual partitions in digital PCR nanoplates (Qiagen QIAcuity) for the *mexB* assay. In addition to the positive and negative partition clusters, many partitions exhibited fluorescence signals within the intermediate region between the two clusters, a pattern commonly referred to as “rain.”

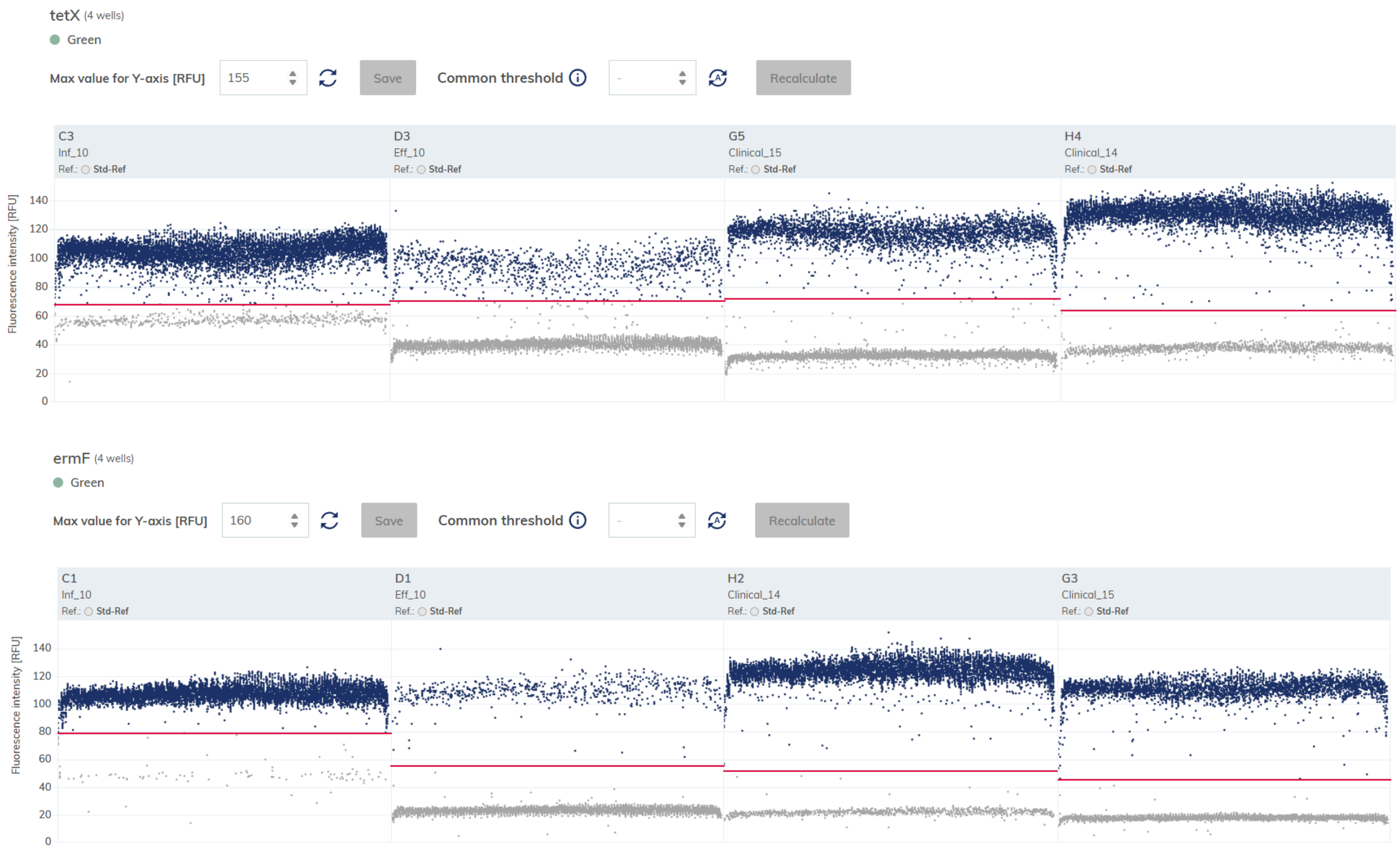

Figure S6. Scatter plots of endpoint fluorescence signals from individual partitions in digital PCR nanoplates (Qiagen QIAcuity) for the *tet(X)* (top) and *ermF* (bottom) assays. Reduced fluorescence intensity was observed in wastewater samples, but not in clinical samples, for the *tet(X)* assay, a pattern commonly associated with PCR inhibition. In contrast, a similar pattern was not observed for the *ermF* assay. The same wastewater samples (Inf_10 and Eff_10) and clinical samples (Clinical_14 and Clinical_15) were used for both assays.

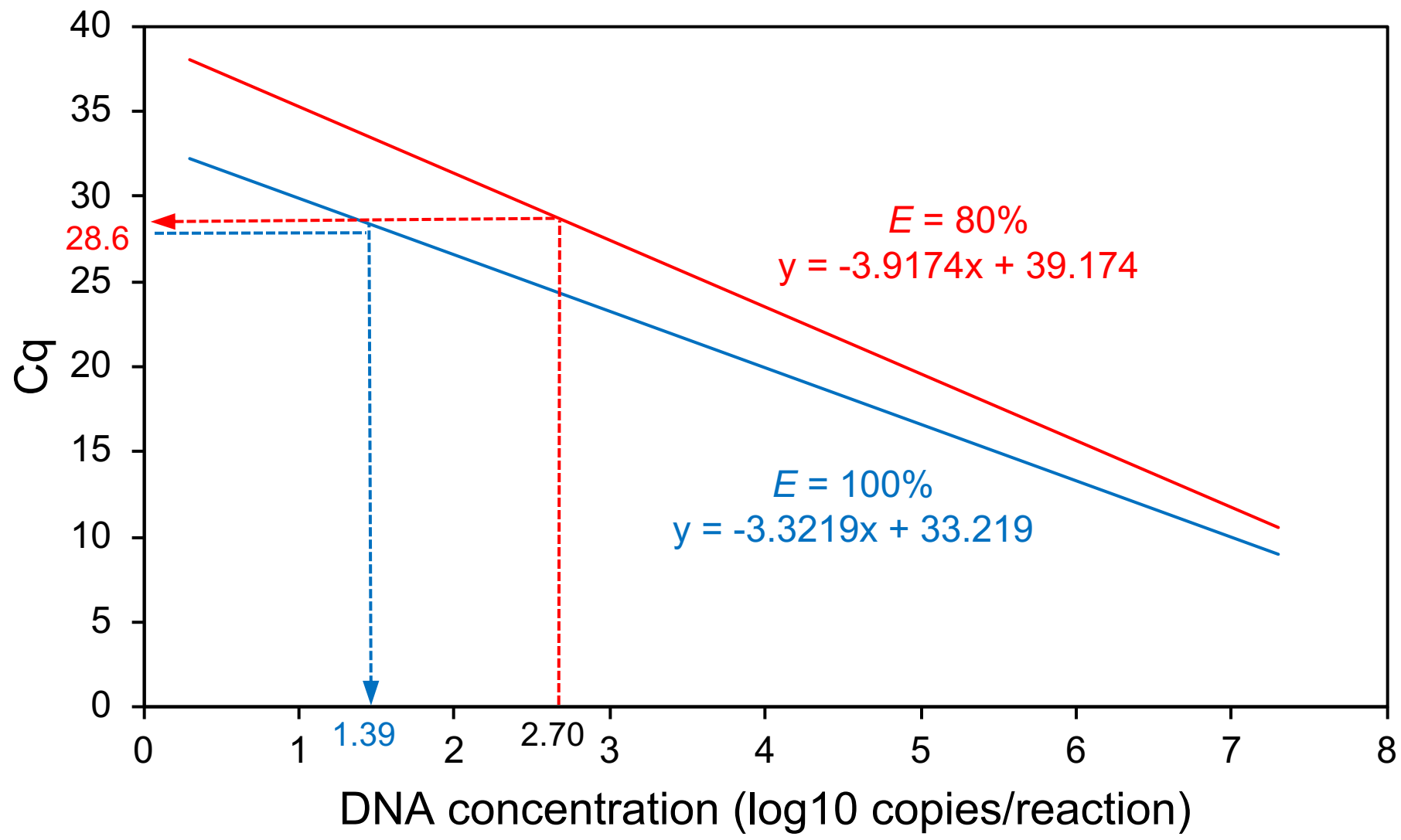

Figure S7. Theoretical standard curves with different amplification efficiencies (*E*). Standard curves were generated by solving Eq. 1 below (Ref. 67) for various initial DNA concentrations (N_0), ranging between 2×10^0 and 2×10^7 copies/μL. C_q is a quantification cycle (also known as a threshold cycle C_T), and N_q is the number of amplicons (copies/reaction) generated at the C_q cycle. The N_q of 10^{10} copies/reaction is used in this calculation.

$$Cq = \frac{\log_{10} N_q}{\log_{10}(1+E)} - \frac{1}{\log_{10}(1+E)} \times \log_{10} N_0 \qquad \text{(Eq. 1)}$$

Assume the concentration of a target gene in a complex environmental (or fecal) DNA sample is 500 copies/reaction (= 2.70 log copies/reaction). The amplification efficiency in these samples can be low (e.g. 80%) due to PCR inhibition and competition between target and non-target amplification. The C_q value to detect 500 copies/reaction (= 2.70 log copies/reaction) is 28.6 based on the standard curve for *E* = 80% shown in this figure (see the red dashed line). However, the amplification efficiency for pure DNA standards (e.g. gBlock DNA fragments) can be near 100%. If the DNA concentration of an environmental sample is estimated using the standard curve for *E* = 100% with a Cq value of 28.6, it gives 24.6 copies/reaction (1.39 log copies/reaction) (see the blue dashed line), significantly underestimating the true concentration (500 copies/reaction = 2.70 log copies/reaction).

REFERENCES

1. Layton A, McKay L, Williams D, Garrett V, Gentry R, Sayler G. 2006. Development of *Bacteroides* 16S rRNA gene TaqMan-based real-time PCR assays for estimation of total, human, and bovine fecal pollution in water. *Appl Environ Microbiol 72*:4214-4224.
2. Lee D-Y, Weir SC, Lee H, Trevors JT. 2010. Quantitative identification of fecal water pollution sources by TaqMan real-time PCR assays using *Bacteroidales* 16S rRNA genetic markers. *Appl Microbiol Biotechnol* 88:1373-1383.
3. Green HC, Haugland RA, Varma M, Millen HT, Borchardt MA, Field KG, Walters WA, Knight R, Sivaganesan M, Kelty CA, Shanks OC. 2014. Improved HF183 quantitative real-time PCR assay for characterization of human fecal pollution in ambient surface water samples. *Appl Environ Microbiol* 80:3086-3094.
4. Lee CS, Lee J. 2010. Evaluation of new *gyrB*-based real-time PCR system for the detection of *B. fragilis* as an indicator of human-specific fecal contamination. *J Microbiol Methods* 82:311-318.
5. Shanks OC, Kelty CA, Sivaganesan M, Varma M, Haugland RA. 2009. Quantitative PCR for genetic markers of human fecal pollution. *Appl Environ Microbiol* 75:5507-5513.
6. Gomi R, Matsuda T, Matsui Y, Yoneda M. 2014. Fecal source tracking in water by next-generation sequencing technologies using host-specific *Escherichia coli* genetic markers. *Environ Sci Technol* 48:9616-9623.
7. Senkbeil JK, Ahmed W, Conrad J, Harwood VJ. 2019. Use of *Escherichia coli* genes associated with human sewage to track fecal contamination source in subtropical waters. *Sci Total Environ* 686:1069-1075.
8. Johnston C, Ufnar JA, Griffith JF, Gooch JA, Stewart JR. 2010. A real-time qPCR assay for the detection of the *nifH* gene of *Methanobrevibacter smithii*, a potential indicator of sewage pollution. *J Appl Microbiol* 109:1946-1956.
9. Stachler E, Kelty C, Sivaganesan M, Li X, Bibby K, Shanks OC. 2017. Quantitative CrAssphage PCR assays for human fecal pollution measurement. *Environ Sci Technol* 51:9146-9154.
10. Caldwell JM, Raley ME, Levine JF. 2007. Mitochondrial multiplex real-time PCR as a source tracking method in fecal-contaminated effluents. *Environ Sci Technol* 41:3277-3283.
11. Kildare BJ, Leutenegger CM, McSwain BS, Bambic DG, Rajal VB, Wuertz S. 2007. 16S rRNA-based assays for quantitative detection of universal, human-, cow-, and dog-specific fecal *Bacteroidales*: A Bayesian approach. *Water Res* 41:3701-3715.
12. Dick LK, Bernhard AE, Brodeur TJ, Santo Domingo JW, Simpson JM, Walters SP, Field KG. 2005. Host distributions of uncultivated fecal *Bacteroidales* bacteria reveal genetic markers for fecal source identification. *Appl Environ Microbiol* 71:3184-3191.
13. Sinigalliano CD, Fleisher JM, Gidley ML, Solo-Gabriele HM, Shibata T, Plano LRW, Elmir SM, Wanless D, Bartkowiak J, Boiteau R, Withum K, Abdelzaher AM, He G, Ortega C, Zhu X, Wright ME, Kish J, Hollenbeck J, Scott T, Backer LC, Fleming LE. 2010. Traditional and molecular analyses for fecal indicator bacteria in non-point source subtropical recreational marine waters. *Water Res* 44:3763-3772.
14. Caldwell JM, Levine JF. 2009. Domestic wastewater influent profiling using mitochondrial real-time PCR for source tracking animal contamination. *J Microbiol Methods* 77:17-22.
15. Shanks OC, Atikovic E, Blackwood AD, Lu J, Noble RT, Santo Domingo J, Seifring S, Sivaganesan M, Haugland RA. 2008. Quantitative PCR for detection and enumeration of genetic markers of bovine fecal pollution. *Appl Environ Microbiol* 74:745-752.
16. Reischer GH, Kasper DC, Steinborn R, Mach RL, Farnleitner AH. 2006. Quantitative PCR method for sensitive detection of ruminant fecal pollution in freshwater and evaluation of this method in alpine karstic regions. *Appl Environ Microbiol* 72:5610-5614.

17. Mieszkin S, Yala J-F, Joubrel R, Gourmelon M. 2010. Phylogenetic analysis of *Bacteroidales* 16S rRNA gene sequences from human and animal effluents and assessment of ruminant faecal pollution by real-time PCR. *J Appl Microbiol* 108:974-984.
18. Mieszkin S, Furet JP, Corthier G, Gourmelon M. 2009. Estimation of pig fecal contamination in a river catchment by real-time PCR using two pig-specific *Bacteroidales* 16S rRNA genetic markers. *Appl Environ Microbiol* 75:3045-3054.
19. Ohad S, Ben-Dor S, Prilusky J, Kravitz V, Dassa B, Chalifa-Caspi V, Kashi Y, Rorman E. 2016. The development of a novel qPCR assay-set for identifying fecal contamination originating from domestic fowls and waterfowl in Israel. Front. Microbiol. 7:145.
20. Ludwig W, Schleifer K-H. 2000. How quantitative is quantitative PCR with respect to cell counts? *Syst Appl Microbiol* 23:556-562.
21. Chern EC, Brenner KP, Wymer L, Haugland RA. 2009. Comparison of fecal indicator bacteria densities in marine recreational waters by QPCR. *Water Qual Expo Health* 1:203-214.
22. Sharma VK, Dean-Nystrom EA, Casey TA. 1999. Semi-automated fluorogenic PCR assays (TaqMan) for rapid detection of *Escherichia coli* O157:H7 and other Shiga toxigenic *E. coli*. *Mol Cell Probes* 13:291-302.
23. Hsu C-F, Tsai T-Y, Pan T-M. 2005. Use of the duplex TaqMan PCR system for detection of Shiga-like toxin-producing *Escherichia coli* O157. *J Clin Microbiol* 43:2668-2673.
24. Thiem VD, Sethabutr O, von Seidlein L, Van Tung T, Canh DG, Chien BT, Tho LH, Lee H, Houng H-S, Hale TL, Clemens JD, Mason C, Trach DD. 2004. Detection of *Shigella* by a PCR assay targeting the *ipaH* gene suggests increased prevalence of shigellosis in Nha Trang, Vietnam. *J Clin Microbiol* 42:2031-2035.
25. Siala, M, Rihl, M, Sellami, H, Znazen, A, Sassi, N, Laadhar, L, Gdoura, R, Belghuith, I, Mrabet, D, Baklouti, S, Sellami, S, Sibilia, J, Fourati, H, Hammami, A, Cheour, I. 2018. Detection of *Shigella* spp. nucleic acids in the synovial tissue of Tunisian rheumatoid arthritis patients and other forms of arthritis by quantitative real-time polymerase chain reaction. *Rheumatol Int* 38:1009-1016.
26. González-Escalona N, Hammack TS, Russell M, Jacobson AP, De Jesús AJ, Brown EW, Lampel KA. 2009. Detection of live *Salmonella* sp. cells in produce by a TaqMan-based quantitative reverse transcriptase real-time PCR targeting *invA* mRNA. *Appl Environ Microbiol* 75:3714-3720.
27. Malorny B, Paccassoni E, Fach P, Bunge C, Martin A, Helmuth R. 2004. Diagnostic real-time PCR for detection of *Salmonella* in food. *Appl Environ Microbiol* 70:7046-7052.
28. Poli VFS, Thorsen L, Olesen I, Wik MT, Jespersen L. 2012. Differentiation of the virulence potential of *Campylobacter jejuni* strains by use of gene transcription analysis and a Caco-2 assay. *Int J Food Microbiol* 155:60-68.
29. Toplak N, Kovač M, Piskernik S, Možina SS, Jeršek B. 2012. Detection and quantification of *Campylobacter jejuni* and *Campylobacter coli* using real-time multiplex PCR. *J Appl Microbiol* 112:752-764.
30. Gurjar AA, Hegde NV, Love BC, Jayarao BM. 2008. Real-time multiplex PCR assay for rapid detection and toxintyping of *Clostridium perfringens* toxin producing strains in feces of dairy cattle. *Mol Cell Probes* 22:90-95.
31. Wise MG, Siragusa GR. 2005. Quantitative detection of *Clostridium perfringens* in the broiler fowl gastrointestinal tract by real-time PCR. *Appl Environ Microbiol* 71:3911-3916.
32. Chen Y, Kumar N, Siddique N. 2011. Development and evaluation of a real-time polymerase chain reaction assay targeting *iap* for the detection of *Listeria monocytogenes* in select food matrices. *Foodborne Pathog Dis* 8:1063-1069.
33. Suo B, He Y, Tu S-I, Shi X. 2010. A multiplex real-time polymerase chain reaction for simultaneous detection of *Salmonella* spp., *Escherichia coli* O157, and *Listeria monocytogenes* in meat products. *Foodborne Pathog Dis* 7:619-628.

34. Blackstone GM, Nordstrom JL, Bowen MD, Meyer RF, Imbro P, DePaola A. 2007. Use of a real time PCR assay for detection of the *ctxA* gene of *Vibrio cholerae* in an environmental survey of Mobile Bay. *J Microbiol Methods* 68:254-259.
35. Liu J, Gratz J, Maro A, Kumburu H, Kibiki G, Taniuchi M, Howlader AM, Sobuz SU, Haque R, Talukder KA, Qureshi S, Zaidi A, Haverstick DM, Houpt ER. 2012. Simultaneous detection of six diarrhea-causing bacterial pathogens with an in-house PCR-Luminex assay. *J Clin Microbiol* 50:98-103.
36. Liu J, Gratz J, Amour C, Kibiki G, Becker S, Janaki L, Verweij JJ, Taniuchi M, Sobuz SU, Haque R, Haverstick DM, Houpt ER. 2013. A laboratory-developed TaqMan Array Card for simultaneous detection of 19 enteropathogens. *J Clin Microbiol* 51:472-480.
37. Blackstone GM, Nordstrom JL, Vickery MCL, Bowen MD, Meyer RF, DePaola A. 2003. Detection of pathogenic *Vibrio parahaemolyticus* in oyster enrichments by real time PCR. *J Microbiol Methods* 53:149-155.
38. Benitez AJ, Winchell JM. 2013. Clinical application of a multiplex real-time PCR assay for simultaneous detection of *Legionella* species, *Legionella pneumophila*, and *Legionella pneumophila* serogroup 1. *J Clin Microbiol* 51:348-351.
39. Radomski N, Roguet A, Lucas FS, Veyrier FJ, Cambau E, Accrombessi H, Moilleron R, Behr MA, Moulin L. 2013. *atpE* gene as a new useful specific molecular target to quantify *Mycobacteriumin* environmental samples. *BMC Microbiol* 13:277.
40. Rocchetti TT, Silbert S, Gostnell A, Kubasek C, Widen R. 2016. Validation of a multiplex real-time PCR assay for detection of *Mycobacterium* spp. *Mycobacterium tuberculosis* complex, and *Mycobacterium avium* complex directly from clinical samples by use of the BD Max Open System. *J Clin Microbiol* 2016, 54:1644-1647.
41. Guy RA, Payment P, Krull UJ, Horgen PA. 2003. Real-time PCR for quantification of *Giardia* and *Cryptosporidium* in environmental water samples and sewage. *Appl Environ Microbiol* 69:5178-5185.
42. Roy S, Kabir M, Mondal D, Ali IKM, Petri WA, Haque R. 2005. Real-time-PCR assay for diagnosis of *Entamoeba histolytica* infection. *J Clin Microbiol* 43:2168-2172.
43. Qvarnstrom Y, Visvesvara GS, Sriram R, da Silva AJ. 2006. Multiplex real-time PCR assay for simultaneous detection of *Acanthamoeba* spp. *Balamuthia mandrillaris*, and *Naegleria fowleri*. *J Clin Microbiol* 44:3589-3595.
44. Szczepanowski R, Linke B, Krahn I. 2009. Detection of 140 clinically relevant antibiotic-resistance genes in the plasmid metagenome of wastewater treatment plant bacteria showing reduced susceptibility to selected antibiotics. *Microbiology* 155:2306-2319.
45. Hindiyeh M, Smollen G, Grossman Z. 2008. Rapid detection of bla_{KPC} carbapenemase genes by real-time PCR. *J Clin Microbiol* 46:2879-2883.
46. Ahammad ZS, Sreekrishnan TR, Hands CL, Knapp CW, Graham DW. 2014. Increased waterborne bla_{NDM-1} resistance gene abundances associated with seasonal human pilgrimages to the Upper Ganges River. *Environ Sci Technol* 48:3014-3020.
47. Ross J, Topp E. 2015. Abundance of antibiotic resistance genes in bacteriophage following soil fertilization with dairy manure or municipal biosolids, and evidence for potential transduction. *Appl Environ Microbiol* 81:7905-7913.
48. Chia JH, Chu C, Su LH. 2005. Development of a multiplex PCR and SHV melting-curve mutation detection system for detection of some SHV and CTX-M β-lactamases of *Escherichia coli*, *Klebsiella pneumoniae*, and *Enterobacter cloacae* in Taiwan. *J Clin Microbiol* 43:4486-4491.
49. Taghavi S, Lesaulnier C, Monchy S. 2009. Lead(II) resistance in *Cupriavidus metallidurans* CH34: interplay between plasmid and chromosomally-located functions. *Antonie van Leeuwenhoek* 96:171–182.

50. Patra RC, Malik S, Beer M. 2010. Molecular characterization of chromium (VI) reducing potential in Gram positive bacteria isolated from contaminated sites. *Soil Biol Biochem* 42:1857-1863.
51. Shahla SN, Mohammad RK, Giti E. 2014. Molecular analysis of copper resistance determinant (*copA*) in copper oxide nanoparticles resistant *Pseudomonas fluorescens* CuO-2 isolated from soil. *Intl J Biosci* 5:97-104.
52. Chen J, Yu Z, Michel Jr. FC, Wittum T, Morrison M. 2007. Development and application of real-time PCR assays for quantification of *erm* genes conferring resistance to macrolides-lincosamides-streptogramin B in livestock manure and manure management system. *Appl Environ Microbiol* 73:4407-4416.
53. Ma Y, Wilson CA, Novak JT, Riffat R, Aynur S, Murthy S, Pruden A. 2011. Effect of various sludge digestion conditions on sulfonamide, macrolide, and tetracycline resistance genes and class I integrons. *Environ Sci Technol* 45:7855-7861.
54. Goldstein C, Lee MD, Sanchez S, Hudson C, Phillips B, Register B, Grady M, Liebert C, Summers AO, White DG, Maurer JJ. 2001. Incidence of class 1 and 2 integrases in clinical and commensal bacteria from livestock, companion animals, and exotics. *Antimicrob Agents Chemother* 45:723-726.
55. Barraud O, Baclet MC, Denis F. 2010. Quantitative multiplex real-time PCR for detecting class 1, 2 and 3 integrons. *J Antimicrob Chemother* 65:1642-1645.
56. Pitkanen LK, Tamminen M, Hynninen A. 2001. Fish farming affects the abundance and diversity of the mercury resistance *merA* in marine sediments. *Microbes Environ* 26:205-211.
57. McNamara P, LaPara T, Novak P. 2014. The impacts of triclosan on anaerobic community structures, function, and antimicrobial resistance. *Environ Sci Technol* 48:7393-7400.
58. Fantino JR, Py B, Fontecase M. 2010. A genetic analysis of the response of *Escherichia coli* to cobalt stress. *Environ Microbiol* 12:2846–2857.
59. Cummings DE, Archer KA, Arriola DJ. 2011. Broad dissemination of plasmid-mediated quinolone resistance genes in sediments of two urban coastal wetlands. *Environ Sci Technol* 45:447-454.
60. Heuer H, Smalla K. 2007. Manure and sulfadiazine synergistically increased bacterial antibiotic resistance in soil over at least two months. *Environ Microbiol* 9:657-666.
61. Pei R, Kim S-C, Carlson KH, Pruden A. 2006. Effect of river landscape on the sediment concentrations of antibiotics and corresponding antibiotic resistance genes (ARG). *Water Res* 40:2427-2435.
62. Ng L-K, Martin I, Alfa M, Mulvey M. 2001. Multiplex PCR for the detection of tetracycline resistant genes. *Mol Cell Probes* 15:209-215.
63. Aminov RI, Garrigues-Jeanjean N, Mackie RI. 2001. Molecular ecology of tetracycline resistance: Development and validation of primers for detection of tetracycline resistance genes encoding ribosomal protection proteins. *Appl Environ Microbiol* 67:22-32.
64. Ghosh S, Sadowsky MJ, Roberts MC, Gralnick JA, LaPara TM. 2009. *Sphingobacterium* sp. strain PM2-P1-29 harbours a functional *tet(X)* gene encoding for the degradation of tetracycline. *J Appl Microbiol* 106:1336-1342.
65. Bell JM, Paton JC, Turnidge J. 1998. Emergence of vancomycin-resistant enterococci in Australia: Phenotypic and genotypic characteristics of isolates. *J Clin Microbiol* 36:2187-2190.
66. Graham M, Ballard A, Grabasch EA. 2008. High rates of fecal carriage of nonenterococcal *vanB* in both children and adults. *Antimicrob Agents Chemother* 52: 1195-1197.
67. Schmidt PJ, Acosta N, Chik AHS, D'Aoust PM, Delatolla R, Dhiyebi HA, Glier MB, Hubert CRJ, Kopetzky J, Mangat CS, Pang X-L, Peterson SW, Prystajecky N, Qiu Y,

Servos MR, Emelko MB. 2023. Realizing the value in “non-standard” parts of the qPCR standard curve by integrating fundamentals of quantitative microbiology. *Front Microbiol* 14:1048661
